# Lake regionalization and diatom metacommunity structuring in tropical South America

**DOI:** 10.1002/ece3.4305

**Published:** 2018-07-13

**Authors:** Xavier Benito, Sherilyn C. Fritz, Miriam Steinitz‐Kannan, Maria I. Vélez, Michael M. McGlue

**Affiliations:** ^1^ Department of Earth and Atmospheric Sciences and School of Biological Sciences University of Nebraska–Lincoln Lincoln Nebraska; ^2^ Department of Biological Sciences Northern Kentucky University Highland Heights Kentucky; ^3^ Department of Geology University of Regina Regina SK Canada; ^4^ Department of Earth and Environmental Sciences University of Kentucky Lexington Kentucky

**Keywords:** diatom guilds, lakes, latitudinal gradient, metacommunity, topographic heterogeneity

## Abstract

Lakes and their topological distribution across Earth's surface impose ecological and evolutionary constraints on aquatic metacommunities. In this study, we group similar lake ecosystems as metacommunity units influencing diatom community structure. We assembled a database of 195 lakes from the tropical Andes and adjacent lowlands (8°N–30°S and 58–79°W) with associated environmental predictors to examine diatom metacommunity patterns at two different levels: taxon and functional (deconstructed species matrix by ecological guilds). We also derived spatial variables that inherently assessed the relative role of dispersal. Using complementary multivariate statistical techniques (principal component analysis, cluster analysis, nonmetric multidimensional scaling, Procrustes, variance partitioning), we examined diatom–environment relationships among different lake habitats (sediment surface, periphyton, and plankton) and partitioned community variation to evaluate the influence of niche‐ and dispersal‐based assembly processes in diatom metacommunity structure across lake clusters. The results showed a significant association between geographic clusters of lakes based on gradients of climate and landscape configuration and diatom assemblages. Six lake clusters distributed along a latitudinal gradient were identified as functional metacommunity units for diatom communities. Variance partitioning revealed that dispersal mechanisms were a major contributor to diatom metacommunity structure, but in a highly context‐dependent fashion across lake clusters. In the Andean Altiplano and adjacent lowlands of Bolivia, diatom metacommunities are niche assembled but constrained by either dispersal limitation or mass effects, resulting from area, environmental heterogeneity, and ecological guild relationships. Topographic heterogeneity played an important role in structuring planktic diatom metacommunities. We emphasize the value of a guild‐based metacommunity model linked to dispersal for elucidating mechanisms underlying latitudinal gradients in distribution. Our findings reveal the importance of shifts in ecological drivers across climatic and physiographically distinct lake clusters, providing a basis for comparison of broad‐scale community gradients in lake‐rich regions elsewhere. This may help guide future research to explore evolutionary constraints on the rich Neotropical benthic diatom species pool.

## 1 INTRODUCTION

Lakes are one focal point in biogeography and community ecology, because they cover a small proportion of the Earth's surface but make disproportionate contributions to regional biodiversity, global biogeochemical cycles, and ecosystem services (Vitousek, Mooney, Lubchenco, & Melillo, 1997). The overlap between biogeography and community ecology is rapidly expanding, particularly for questions that examine the effects of spatiotemporal gradients and the influences of historical legacies (dispersal) on contemporary regional and local biotas (Jenkins & Ricklefs, 2011; Verleyen et al., 2009; Vyverman et al., 2007). In this context, metacommunity theory, which recognizes a set of local ecological communities that are connected by the dispersal of potentially interacting species, provides a flexible framework to integrate both disciplines, because it considers both regional (e.g., dispersal, climate) and local (e.g., biotic interactions, limnology) drivers of species composition (Gonçalves‐Souza, Romero, & Cottenie, 2014; Leibold et al., 2004; Soininen, Jamoneau, Rosebery, & Passy, 2016; Viana et al., 2014). Combining biogeography and metacommunity theory is timely in light of global environmental change, because impacts from climate change (global processes) to habitat fragmentation and pollution (local processes) will likely affect lake ecosystems at intermediate spatial scales (Jenkins & Ricklefs, 2011).

Implicitly, metacommunity theory focuses on two nonexclusive paradigms to explain the composition of ecological communities: species sorting, with an emphasis on species autoecology and environmental gradients as the major influences (niche assembly rules), and dispersal (Heino et al., 2015). Species sorting requires a moderate dispersal rate to allow species to be sorted into their most suitable environmental habitats. When dispersal rates are high, communities are homogenized irrespective of environmental conditions via mass effects, thereby obscuring species sorting, particularly at small spatial scales. In contrast, low dispersal rates hinder a species ability to effectively track suitable environmental conditions, resulting in dispersal limited communities, especially at large spatial extents (Heino et al., 2015). Recent metacommunity studies propose that ecological drivers of community structure should fit within a framework defined by environmental heterogeneity, spatial scale, and the innate dispersal abilities of the organisms themselves (Brown, Sokol, Skelton, & Tornwall, 2017; Passy, 2017).

For organisms that disperse widely and passively, like microalgae or fungi, few studies have used a deconstructive approach that splits community data into different functional groups as surrogates for dispersal ability (Bie et al., 2012). Diatoms (unicellular siliceous algae) are useful organisms to study, because they are a very species‐rich group of algae, disperse widely, and have different growth morphologies for resource use and to resist physical disturbances (Passy, 2017). Passy (2007) and Rimet and Bouchez, (2011) classified diatom species into four ecological guilds: high‐profile, low‐profile, motile, and planktic, with all guilds supposedly connected to their dispersal abilities (Wetzel et al., 2012). Recent findings indicate that environmental and spatial mechanisms that structure species composition differ across guilds in lotic diatoms (Dong et al., 2016; Liu, Soininen, Han, & Declerck, 2013; Soininen et al., 2016; Wetzel et al., 2012), yet little evidence exists for process that structure diatom species composition in lakes. For instance, Vilmi, Tolonen, Karjalainen, and Heino (2017) hypothesized that diatom species tightly attached to the substratum, typically corresponding to the high‐profile and low‐profile guilds, are less likely to be dispersed than species that can move along a substrate or are floating in the water column, such as the motile and planktic guilds, respectively. However, no previous study has analyzed diatom metacommunity structuring across gradients of climate, physiography, and limnology in the tropics, and certainly not using a trait‐based approach. Thus, we apply this approach to lakes of tropical South America, where very diverse benthic and planktic floras and many lake‐rich regions are present (Rumrich, Lange‐Bertalot, & Rumrich, 2000). Besides the strong provinciality observed in the Southern Hemisphere diatom flora (Vyverman et al., 2007), tropical lakes have neither been previously compared nor analyzed to explain possible mechanisms behind diversity gradients.

From a landscape perspective, lake ecosystems are units defined by the surrounding catchment, including topography, land cover, bedrock geology, and climate. Lakes are rarely isolated on the landscape; clusters or lake districts are the rule, not the exception (Catalan, Curtis, & Kernan, 2009). This makes lakes ideal systems to test macroecological effects of local and regional processes on assemblage composition (i.e., “biogeographic islands,” Colinvaux & Steinitz‐Kannan, 1980). Disentangling the relative effects of pure environmental versus spatial factors on community species composition using empirical data is generally challenging, partly because of the presence of many direct and indirect connections among landscape processes operating at different spatial and temporal scales (Logue, Mouquet, Peter, & Hillebrand, 2011). Moreover, from the high mountains to lowlands, a diversity of conditions associated with topography (e.g., slope, aspect etc.) and continuum of aquatic systems impose a variety of dispersal pathways in organisms (e.g., geographic, network, landscape resistance) (Moritz et al., 2013). Thus, the ecological consequences of lake districts for metacommunities are not well understood (Catalan & Donato Rondon, 2016).

**Figure 1:**
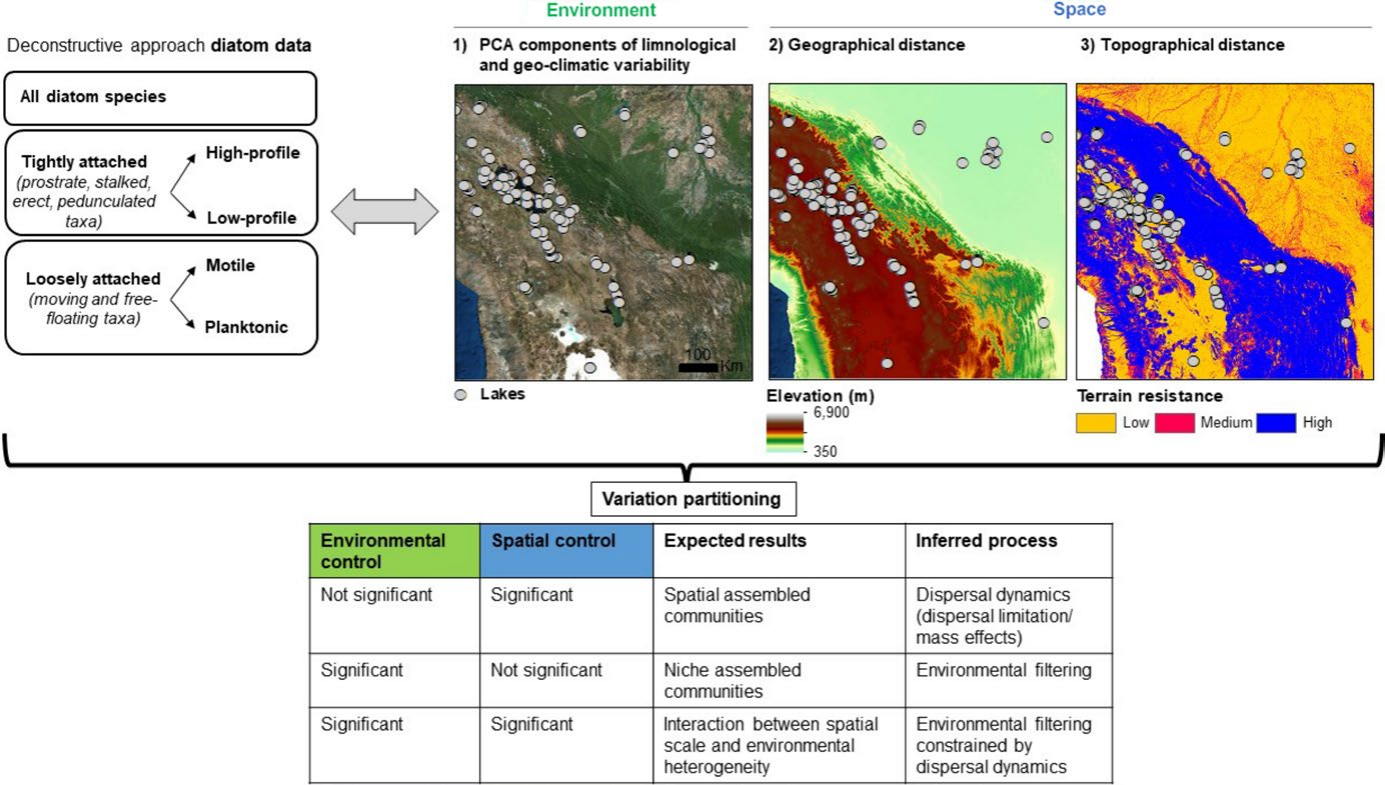
Study design and working hypothesis to examine lacustrine diatom metacommunity patterns in tropical South America. Diatom data were analyzed using taxon and functional approaches (deconstructed species matrix by ecological guilds). We used three sets of predictors that represent environment, geographic, and topographic components to investigate the influence of niche and dispersal effects on diatom community structure. Variation partitioning analysis was used to quantify pure and shared proportions of variation on community composition explained by the three set of predictors. We summarized inferred processes according to variation partitioning results and the expected results of environmental and spatial controls

The overarching aim of this study is to classify groups of tropical lakes as functional units to examine diatom metacommunity structuring. First, we examined the extent to which limnological and geo‐climatic environmental factors typify groups of lakes and how such classification correlates with diatom distribution. Second, we analyzed the relative importance of environmental and spatial structuring of diatom assemblages for each lake cluster individually. Third, we tested whether environmental and/or spatial processes differed between diatom guilds and lake clusters. Given the link between life forms and dispersal abilities of organisms in metacommunity structuring, we hypothesize that diatom guilds tightly attached to the substratum (high‐profile and low‐profile, “weak dispersers”) are strongly affected by spatial variables, whereas guilds that glide along substrates or are free floating (motile and planktic, “strong dispersers”) are strongly influenced by environmental variables (e.g., water chemistry, climate) (Figure 1).

## 2 MATERIAL AND METHODS

### 2.1 Study area

**Figure 2:**
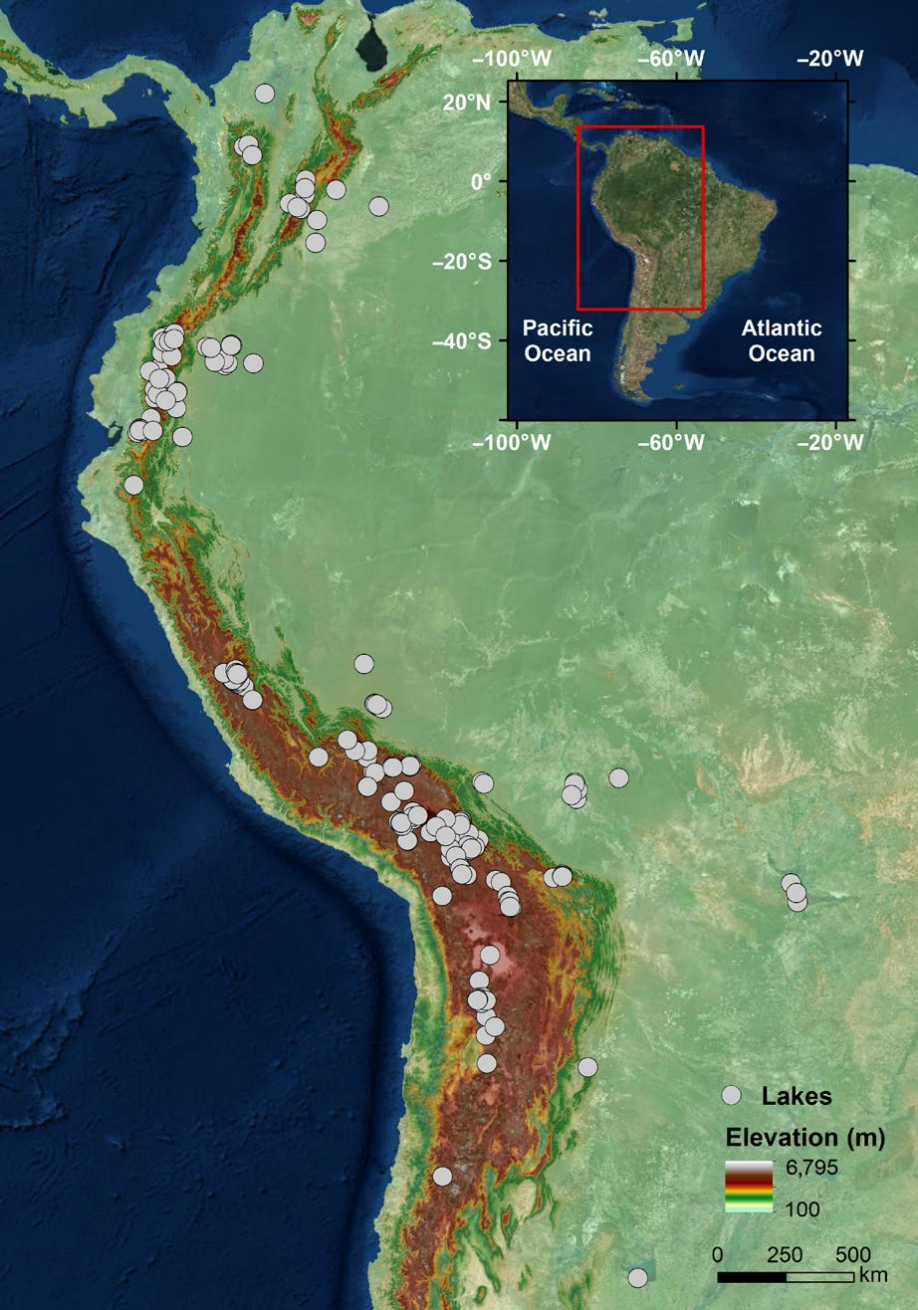
Map of tropical South America showing the location of the study lakes (*n* = 195). See Supporting Information Appendix S1—Table S1.1 for detailed information about the lakes

The study lakes are distributed across the tropical Andes and the Andean foreland plains (Figure 2), covering an altitudinal gradient from 220 to 5,070 m a.s.l. between 8°N–30°S and 58–79°W. Our lake database encompasses a wide range of physiographic and climatic settings that produce diverse limnological conditions. Lowland sites are primarily lakes that occupy old river channels and floodplain wetlands, spread across Ecuador, Perú, Bolivia, and Brazil. In these lowland regions, some lake basins are seasonally connected to large rivers (e.g., Parana, Paraguay, Napo), which increases seasonal variability in limnological and hydrological conditions (McGlue et al., 2011). Colombia's lowland lakes are distributed from the eastern savannas to the very wet western rain forests (Vélez, Wille, Hooghiemstral, & Metcalfe, 2005). Along the Andean cordillera, lakes occur at a range of high elevations (3,000–5,070 m a.s.l.) and lie in closed (endorheic) basins. The tropical Andes shows a predominant north–south landscape gradient with varied topographic heterogeneity that influences both local and regional climates (Valencia et al., 2016). Northern Andean lakes in Ecuador and Colombia lie in montane forests, inter‐Andean valleys, and Páramo ecosystems. In the central Andean Cordillera of Perú and Bolivia, most of the study lakes are closed basins of glacial origin dominated by montane grass and shrubland. In the Altiplano plateau (central Andes), the northern region is characterized by cold and relatively humid conditions. Lakes are mainly freshwater and lie in extensive interconnected hinterland basins (Cohen et al., 2014). The southern Altiplano is drier, and most lakes are isolated and saline due to the basin geology and high evaporation rates (Sylvestre, Servant‐Vildary, & Roux, 2001).

### 2.2 Diatom database

Diatom data included 195 lakes with a total of 303 samples (Supporting Information Appendix S1—Table S1.1). Diatom samples comprised sediment surface, periphyton, and plankton, to ensure that the majority of diatom ecological guilds were collected. The samples were collected in the period 1977–2009 and fall well within the temporal window of the analyzed climatic variables for comparison. Diatom samples were analyzed separately for habitat type and sampling year, except for the Ecuador dataset, in which samples were composited. Preliminary analyses showed that little variation was seen between sampling dates. Diatom samples were cleaned using 30% H_2_O_2_ and 37% HCl to remove organic material and carbonates, respectively. Cleaned diatoms were mounted in Naphrax (refractive index 1.74). All identifications were made to the species level when possible, using South American diatom floras and regional studies (Manguin & Manguin, 1964; Metzeltin & Lange‐Bertalot, 2007; Rumrich et al., 2000; Servant‐Vildary, 1986) and taxonomic resources available at the diatom herbarium of the Philadelphia Academy of Natural Sciences. In about 90% of the samples, species relative abundance was enumerated by counting >300 diatom valves; the species data of the remaining 10% of the samples consisted of presence–absence counts. To better account for differences in the sampling methodology and counting methods, site‐by‐species abundance matrices were transformed to site‐by‐species presence–absence matrices prior to all analyses. Measures to ensure taxonomic consistency included aggregating varieties of species, scanning the data for taxonomic synonyms, and lumping species complexes (e.g., *Achnanthidium minutissimum*,* Sellaphora pupula*,* Discostella stelligera*,* Ulnaria ulna*) from the database entries. Taxonomic harmonization was carried out to update diatom taxonomic nomenclature based on the most up‐to‐date classification (Guiry & Guiry, 2017).

### 2.3 Ecological guilds

The diatom species matrix was split into ecological guilds to model the dispersal potential of diatoms taxa following Passy (2007) and Rimet and Bouchez (2011). The four guilds are high‐profile, low‐profile, motile, and planktic. The high‐ and low‐profile guilds were considered to be “weak dispersers,” which includes species tightly attached to the substrate, such as adnate, prostrate, stalked, erect, and pedunculated taxa, such as *Achnanthes*,* Gomphonema*, or *Cocconeis*. High‐profile and low‐profile species are adapted to a certain degree to physical disturbances by growing closely to the substrate; high‐profile species are adapted to high nutrient concentrations, and low‐profile species are adapted to low nutrient concentrations. The motile and planktic guilds were considered to be “strong dispersers” and include species either loosely attached or with no obvious method of attachment, such as *Navicula*,* Nitzschia*,* Cyclostephanos*, and *Discostella*. All guild assignments were made at the genus level where possible (Supporting Information Appendix S3).

### 2.4 Environmental and spatial variables

Different sets of explanatory variables were collected from several sources to characterize local and regional environmental gradients of the study lakes. Local environmental variables included limnological parameters that have been identified as important for lake diatoms in the tropical Andes in previous studies (Benito et al., 2018; Steinitz‐Kannan, 1979; Sylvestre et al., 2001; Tapia, Fritz, Seltzer, Rodbell, & Metiever, 2006) and that were available for a large number of sites. These proximal (i.e., site‐specific) environmental variables consisted of water temperature (°C), pH, and conductivity (μS/cm). Regional environmental variables included geographical and climatic variables. A total of 11 geo‐climatic variables were used, including: latitude/longitude, elevation (m), mean annual air temperature (MAT, °C), mean annual precipitation (MAP, mm), temperature seasonality (standard deviation; °C), precipitation seasonality (coefficient of variation; mm), % aquatic habitat, connectivity, Terrain Ruggedness Index (TRI), and lake area (km^2^). The STRM 90 m digital elevation model (Jarvis, Reuter, Nelson, & Guevara, 2008) was used to obtain elevation and calculate TRI values for each cell using the Raster Terrain Analysis Plugin in QGIS v.2.8.2 (QGIS Development Team, 2013). The TRI quantifies terrain heterogenity (slope) by summarizing the change in elevation within a 3 × 3 pixel cell grid (Riley, 1999). Climatic variables were obtained and extracted from the WorldClim 1.4 database. WorldClim contains averaged monthly climate data for the period 1950–2000 at a 1 km grid resolution (Hijmans, Cameron, Parra, Jones, & Jarvis, 2005). The Global Lakes and Wetlands Database (GLWD, Lehner & Döll, 2004) comprises lakes, rivers, and different wetland types with a surface area of >0.1 km^2^ in the form of a global map of ~1 km precision. Using the GLWD as a basemap, equal grids of 50 km^2^ were created to extract the surface area occupied by freshwater in each grid as proxy of % aquatic habitat, and the density of water bodies in each grid as proxy of connectivity. The surface area of each lake was obtained by digitizing using the ESRI World Imagery layer as a basemap. All maps were manipulated using ArcGIS 10.4.1.

Two different distance matrices were calculated representing the physical distance (geography) and the resistance of the landscape to dispersal (topography) between pairs of sites to evaluate potential dispersal. Geographical distance was calculated using Euclidean distances, based on site coordinates. To obtain the distance matrix related to landscape resistance, TRI values for each cell were reclassified into three different levels of resistance by assigning values of 1 (low terrain rugosity), 50 (medium terrain rugosity), and 100 (high terrain rugosity) using ArcGis 10.4.1. These resistances were chosen assuming that lake basins with low rugosity are prone to landscape permeability, whereas less connectivity is expected in landscapes with complex topography (high terrain heterogeneity). Next, the resultant raster map was imported into the CIRCUITSCAPE program (McRae, 2006) to calculate pairwise landscape resistance distances to dispersal. CIRCUITSCAPE uses circuit theory to integrate dispersal through grid cells, allowing for multiple pathways between sites.

Spatial variables were generated for each of the two distance matrices (geographic and topographic) using distance‐based Moran's Eigenvector Maps (Dray, Legendre, & Peres‐Neto, 2006). These spatial db‐MEM variables were obtained using principal coordinates of neighborhood matrix (PCNM). This method describes the spatial variability across study sites by generating eigenvector‐based variables, which can be used as predictors in constrained ordination analysis. Only positive eigenvectors were employed as spatial predictors for posterior statistical analysis (variance partitioning). This analysis was performed using the *pcnm* function of the *vegan* package written in R (Oksanen et al., 2016; R Development Team, 2016).

### 2.5 Statistical analyses

All statistical analyses were performed using the R software version 3.3.1 (R Development Team, 2016).

First, we summarized major patterns among limnological and geo‐climatic variability to classify groups of lakes. Prior to running ordination analyses, all variables were transformed (log10[*x* + 1]) to meet assumptions of linearity and homogeneity of variances (homoscedasticity). A principal component analysis (PCA) with the algorithm NIPALS (nonlinear estimation by iterative partial least squares) was performed using the *nipals* function of the *ade4* package (Dray & Dufour, 2007). The NIPALS algorithm allowed the computation of a PCA without deleting samples with missing data or estimating the missing values (Ibáñez et al., 2012). With this approach, we avoid eliminating sites with missing values, because of few widespread measurements of some limnologic variables (see Supporting Information Appendix S1—Table S1.2 for % data values). A previous study of the region, Benito et al. (2018) showed that water temperature and climatic variables (MAP, MAT) are correlated at the spatial scale of the data used for this study. Also, other limnological variables (conductivity, pH, and nutrients) were outperformed by macroecological gradients associated with distinct climatic and topographic conditions, because local environmental conditions are temporally unstable compared with geo‐climatic variables, such as elevation, catchment geology, and ecoregion in lakes of tropical Andes and adjacent lowlands (Benito et al., 2018). Considering these issues, the PCA axes act as composite variables of environmental drivers of lakes. The number of significant PCA axes, which indicate the nonrandom variability, were selected by broken stick model using the *evplot* function (Borcard, Gillet, & Legendre, 2011). In this case, we retained the first three orthogonal components explaining 72% of total variance (Supporting Information Appendix S4). Metrics for usefulness and sampling adequacy of the data for the PCA were obtained with the Kaiser–Meyer–Olkin index (KMO; critical value >0.70; Dziuban & Shirkey, 1974) and Bartlett's test of sphericity (Budaev, 2010), respectively. The PCA site scores of the first three axes were subsequently used as inputs for hierarchical cluster analysis based on Euclidean distances, with flexible beta as the linkage method using the *agnes* function of the *cluster* package (Maechle, 2012). Both methods (i.e., PCA and cluster analysis) are complementary and helped identify groups of lakes having similar environmental characteristics (cluster analysis) and provided information about the pattern of variation within and between groups in ordination space (PCA).

Second, we analyzed the major structure of the diatom data using a nonmetric multidimensional scaling (NMDS) with Chao distance measure on Hellinger transformed presence–absence matrix, in the *vegan* package. The Chao index was selected to account for unseen shared species and thus to reduce bias in sampling effort among study regions (Colwell, Mao, & Chang, 2004). All diatom taxa (*n* = 1,635), including singletons, were included in the analyses. To aid interpretation of the NMDS axes without incorporating any environmental constraint, limnological and geo‐climatic variables were fitted using the environmental fitting technique with the *envfit* function in *vegan*. Except for the Ecuador data set, additional NMDS analyses were performed for the sediment, periphyton, and plankton samples separately to assess variability of lake habitats and determine any relationship with environmental variables. If geo‐climatic variables are the most important factors for structuring diatom habitat samples, the group division of lakes should be related to the regions, showing clear breakpoints in assemblage composition. In contrast, if limnological variables are more important, site groupings should not be related to the regions, and hence no clear breakpoints in assemblage composition should be observed (e.g., Heino, Soininen, Alahuhta, Lappalainen, & Virtanen, 2016). To account for the possible influence of temporal effects due to the time span of diatom collection, sampling year was included as a variable in the environmental fitting procedure.

Third, we characterized each group of lakes identified by PCA and cluster analysis in terms of their environmental heterogeneity and spatial extent following Tonkin, Death, Muotka, Astorga, and Lytle (2018). Environmental heterogeneity was estimated by calculating the mean distance of each site to the corresponding group centroid using the *betadisper* function of the *vegan* package. For spatial extent, the function *ordihull* in *vegan* was used to enclose all sites that form each cluster, and the relative area was then estimated using the *polygon* function. Differences in environmental heterogeneity among clusters were tested using the *adonis2* function in *vegan* (which uses a permutational multivariate analysis of variance with 999 permutations) (PERMANOVA, Anderson, 2005).

Fourth, to explore the relationships among diatom community distribution and environmental variables and to test the significance of any correlation found, we used the Procrustes and analysis of variance following Lisboa et al. (2014). Procrustes is a correlative multivariate method that assesses species–environment relationships obtained from different unconstrained ordinations. The Procrustean analysis was performed between the NMDS (diatom data) and PCA (environmental data) matrices with the three‐first components of each ordination using the *procrustes* and *protest* functions in *vegan*. The degree of concordance is given by the m^2^ statistic and associated *p* values with 999 permutation tests. Lower values of m^2^, which vary from 0 to 1, indicate greater concordance (Peres‐Neto & Jackson, 2001). If both ordinations (NMDS and PCA) are correlated, diatom distributions should be associated with lake clustering, suggesting regionally distinct communities. To further test this idea, we extracted the residual vector of association between diatom and environmental data, the so‐called procrustean association metric (PAM), using the function *residuals*. Therefore, the statistical difference in PAMs across lake clusters was assessed through an analysis of variance (ANOVA) with the function *aov* at significance level *α* = 0.05.

At last, the relative role of environmental and spatial components on diatom community composition was determined with redundancy analysis (RDA) and variance partitioning analysis. Variance partitioning quantifies pure and shared proportions of variation in community composition explained by different set of predictors (Peres‐Neto, Legendre, Dray, & Borcard, 2006). We used eigenvector‐related variables as predictors: the three‐first PCA axes were used as variables related to niche‐based factors but excluding spatial variables (latitude, longitude, connectivity and TRI), and the two sets of eigenvectors extracted from geographic and topographic distances using the PCNM analysis were used as spatial predictors. The diatom community structure was first regressed onto each set of predictors (environmental, geographic and topographic), including all eigenvectors using global RDA models individually, with the *rda* function in *vegan*, and tested for significance. If the global model was significant, a forward selection procedure was performed using the two stop criteria with the *ordiR2step* function of *vegan*. The forward‐selected variables were posteriorly used for the variance partitioning as explanatory variables using the *varpart* function in *vegan*. The pure effects accounted for by the environmental, geographical, and topographical components were tested using the ANOVA function. We primarily based our results on the adjusted *R* ^2^ values rather than significance alone, because we were interested in quantifying the effect sizes. Variance partitioning analyses were performed for the entire diatom species matrix and separately for each ecological guild across all lake clusters.

## 3 RESULTS

### 3.1 Regionalization of lakes

**Figure 3:**
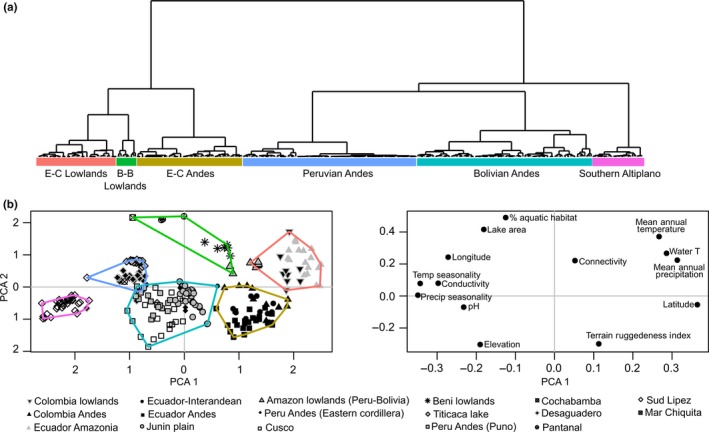
Combined approach of cluster analysis using principal component analysis (PCA) site scores as inputs (a) and PCA ordination with the analyzed limnological and geo‐climatic variables (b). Bottom left: site scores labeled by regions. Bottom right: factor scores of the variables. B‐B: Bolivian‐Brazilian; E‐C: Ecuadorian‐Colombian

The PCA adequately summarizes the limnological and geo‐climatic variability of the data, as indicated by the KMO's measure of adequacy (0.71) and Barlett's test of sphericity (*p *<* *0.001). According to the broken stick model, the amount of non‐random variability is explained by the three‐first principal components, explaining 46.8%, 16.6%, and 10.2%, respectively (Supporting Information Appendix S4); the PCA ordination plot illustrates axes 1 and 2 for graphical purposes (Figure 3b). The first PCA axis is associated with climatic and limnological variability; positive scores occur for MAT, MAP, and water temperature, whereas negative scores were associated with seasonality in precipitation and temperature, pH, and conductivity. The first component arranged lakes from the Amazonian lowlands of Ecuador and Colombia to the central Andes of Bolivia. PCA axis 2 was associated with a lake's landscape configuration (Figure 3b); positive loadings occur for % aquatic habitat, lake area, connectivity, and low elevation, whereas negative scores occurred for high elevation, high terrain heterogeneity, and more isolated conditions (lower % aquatic habitat and connectivity). Thus, the second component separated all lowland sites from the high‐elevation Andean lakes.

Cluster analyses resulted in six lake groups that are arranged along the first PCA axis and exhibit a clear latitudinal structure (Figure 3a and Supporting Information Appendix S2). Environmental heterogeneity differed among the clusters (PERMANOVA: *F *=* *40.537; *p *<* *0.01; Supporting Information Appendix S2—Figure S2.2). Clusters 1 and 2 comprised all lakes near the equator; cluster 1 grouped all lowland lakes (Ecuadorian‐Colombian lowlands, Figure 3a), and cluster 2 grouped Andean lakes from Ecuador and Colombia (Ecuadorian‐Colombian Andes, Figure 3a). Cluster 3 was composed of lakes in the Amazonian lowlands of Bolivia and the Pantanal in Brazil (Bolivian‐Brazilian lowlands, Figure 3a). This cluster showed the highest environmental heterogeneity (Supporting Information Appendix S2—Figure S2.2). Clusters 4, 5, and 6 consisted of high‐elevation Andean lakes across Perú and Bolivia. The Peruvian Andes cluster (cluster 4, Figure 3a) showed a combination of the greatest spatial extent and wide environmental heterogeneity, whereas the Bolivian Andes (cluster 5, Figure 1a) and Southern Altiplano clusters (cluster 6, Figure 3a) had the lowest environmental heterogeneity and spatial extents (Supporting Information Appendix S2—Figure S2).

### 3.2 Diatom metacommunities

Diatom species composition of sediment surface and periphyton samples showed clear regional differences in the NMDS ordination, suggesting that species composition variability is mainly related to among‐region differences (Supporting Information Appendix S5—Figure S5.1–2). In contrast, plankton samples showed less clear separation between regions, suggesting a higher effect of local environmental variables (Supporting Information Appendix S5—Figure S5.3). Based on *R* ^2^ values, geo‐climatic variables had the strongest (*R* ^2^ **> 0.60, *p *<* *0.05) relationship on surface sediment and periphyton species composition, whereas conductivity and pH had the strongest (*R* ^2^ **> 0.65, *p *<* *0.05) relationship on planktic species composition. The relationship between the variable “year” and diatom species composition was statistically significant for the sediment surface (*R* ^2^ **> 0.16; *p *<* *0.05), periphyton (*R* ^2^ **> 0.68; *p *<* *0.05), and plankton (*R* ^2^ **> 0.44; *p *<* *0.05) samples.

**Figure 4:**
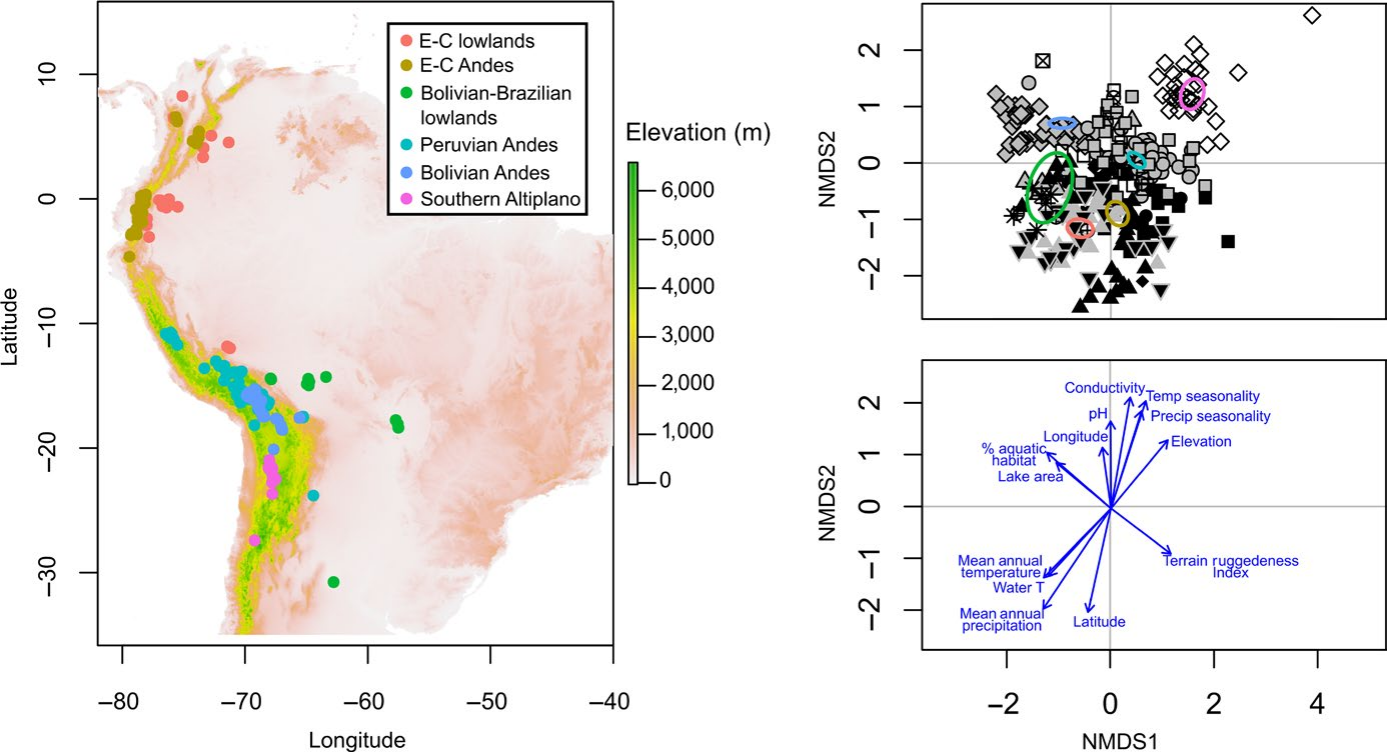
Results of nonmetric multidimensional scaling (NMDS) with Chao distance measure on Hellinger transformed presence–absence diatom data for the entire species matrix (2D stress = 0.20). Left: Lake distribution labeled by clusters. Top right: site scores labeled by regions (see Figure 3 for study region labels); ellipses represent 95% confidence level of each lake's cluster centroid identified through PCA and cluster analyses. Bottom right: environmental variable fitting showing the selected variables; the length of each vector is proportional to the correlation between variables and NMDS axes. E‐C: Ecuadorian‐Colombian

When considering the entire diatom species matrix, limnological and geo‐climatic variables, except for connectivity, had significant relationships with diatom data (*R* ^2^ **> 0.20; *p *<* *0.05), as indicated by the NMDS with environmental fitting (Figure 4). The significant strength of concordance between the NMDS and PCA ordinations (Protest m^2^ statistic = 0.58; *p *<* *0.001) and differences across lake clusters in diatom–environment relationships (ANOVA's PAM: *F *=* *122.3; *p *<* *0.01) supported the cluster division of lakes with distinct diatom communities associated with environmental characteristics of groups of lakes (Figure 4).

**Figure 5:**
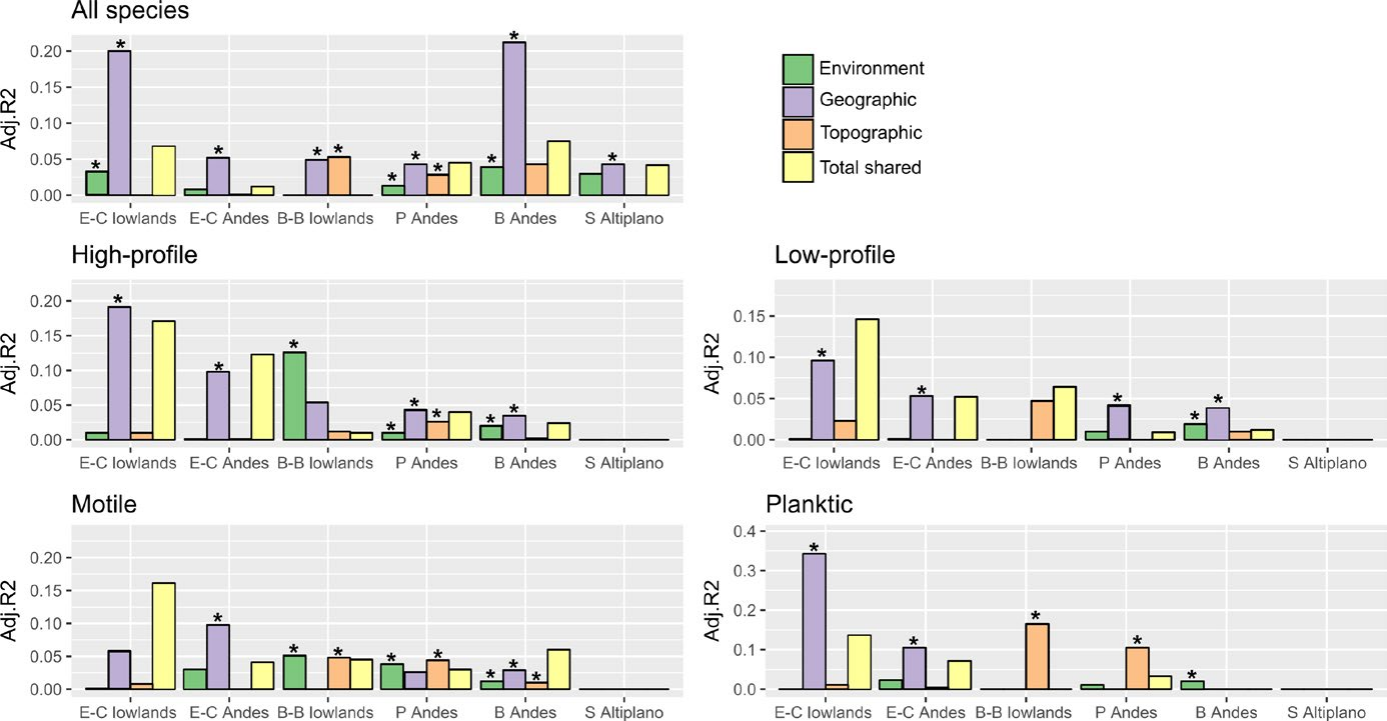
Variance partitioning results of environment, geographic, and topographic variables on all diatom species data and for each guild individually across the six lake clusters identified using PCA and cluster analyses. Asterisks denote the statistical significance (*p *<* *0.05) of the pure fractions of environment, geographic or topographic variables. Lake clusters are arranged from low to high latitude. B: Bolivia; B‐B: Bolivian‐Brazilian; E‐C: Ecuadorian‐Colombian; P: Perú; S: Southern. See Supporting Information Appendix S6—Table S6.1 and Table S6.2 for detailed RDA results and forwarded‐selected variables

The first NMDS axis arranged diatom metacommunities from the lowlands (Ecuadorian‐Colombian lowlands and Bolivian‐Brazilian lowlands, Figure 4) to high‐elevation Andes (Ecuadorian‐Colombian Andes, Peruvian Andes, Bolivian Andes, and Southern Altiplano, Figure 4) following gradients of % aquatic habitat, lake area, and terrain heterogeneity; thus, NMDS axis 1 is primarily a measure of a lake's landscape configuration, similar to PCA axis 2. NMDS axis 2 separated diatom metacommunities from higher latitudes with greater seasonality in precipitation and temperature and higher pH and conductivity (Bolivian‐Brazilian lowlands, Peruvian Andes, Bolivian Andes, and Southern Altiplano, Figure 4), from sites near the equator, where diatom metacommunities were associated with higher MAT and MAP and lower pH and conductivities (Ecuadorian‐Colombian lowlands and Ecuadorian‐Colombian Andes, Figure 5). NMDS axis 2 is thus associated with gradients of climatic and limnological variability, as is PCA axis 1. The variable “year” was significantly correlated with NMDS axis 2 scores (Pearson's *r *=* *0.33; *n* = 370; *p *<* *0.001), but NMDS axis 1 scores were not significantly correlated with this variable (Pearson's *r *=* *−0.11; *n* = 303; *p *=* *0.19).

The RDA results with forward‐selected variables are summarized in Supporting Information Appendix S6. When the whole diatom species matrix is used, variance partitioning revealed that the relative effects of the spatial component (geographic and topographic factors) outperformed the effect of the environmental component in all diatom metacommunities, and equally significant environmental and spatial effects were found in diatom metacommunities for certain high‐latitude lake clusters (Bolivian‐Brazilian lowlands, Peruvian Andes, Bolivian Andes and Southern Altiplano; Figure 5).

If diatom guilds are analyzed individually, varied effects of environmental and spatial components emerged across lake clusters: (a) for the high‐profile and low‐profile guilds (“weak dispersers”), the spatial component overrode the effect of environmental factors in most lake clusters and showed a decreasing trend of the explained variability with latitude (Figure 5); (b) for the motile and planktic guilds (“strong dispersers”), the environmental and spatial components were equally significantly associated with diatom metacommunity structuring in certain lake clusters (Bolivian‐Brazilian lowlands, Peruvian Andes and Bolivian Andes; Figure 5). This was particularly evident for the motile guild (Figure 5), especially for the Bolivian‐Brazilian lowlands, but not for the planktic guild; and (c) for the motile and planktic guilds, the spatial effects on diatom community composition were associated with the topographic component only for certain high‐latitude metacommunities (Bolivian‐Brazilian lowlands and Peruvian Andes), whereas for the low‐latitude metacommunities (Ecuadorean‐Colombian lowlands and Andes), it was associated with the geographic component (Figure 5). The Southern Altiplano showed no environmental or spatial structure for any of the diatom guilds. Of all the diatom guilds, the unique fractions explained by environmental and spatial components were relatively small, and the total shared effect between environmental, geographic, and topographic factors was highest in low‐latitude lake clusters (Figure 5; Supporting Information Appendix S6—Table S6.2).

## 4 DISCUSSION

Our results indicate that lacustrine diatoms in tropical Andean mountains and the adjacent lowlands form ecologically meaningful clusters following gradients of local and regional environmental conditions. The PCA identified two independent driving factors in the formation of lake clusters, namely climatic and limnological variability (PCA1) and landscape configuration (PCA2), which resulted in six different geographically distinct clusters in the way that these environmental factors combine. Covering large environmental gradients and including different within‐lake habitats necessarily crosses multiple species pools that show different response to regional and local environmental factors (Heino et al., 2016). From a diatom community perspective, planktic communities more closely track limnological conditions compared with sediment surface and periphyton communities, suggesting a stronger influence of regional‐scale environmental gradients on benthic diatom species (Winegardner, Beisner, Legendre, & Gregory‐Eaves, 2015). In addition, our study provides new evidence that regional diatom communities can be treated as unique metacommunities, because of the distribution of species in environmentally similar lake clusters, as indicated by the significant association between PCA and NMDS. From a diatom metacommunity perspective, distinctive context‐dependent processes emerged. We argue these processes originate from the relationships between spatial extent, environmental heterogeneity, and ecological guilds (as surrogate of dispersal abilities).

As expected, lake clusters differed in terms of spatial extent and environmental heterogeneity. These two factors are essential for distinguishing among species sorting, dispersal limitation, and mass effects in lake metacommunities (Heino et al., 2015; Vilmi et al., 2017). For instance, a higher environmental heterogeneity within‐lake clusters would lead to stronger species sorting, likely because the clusters have a greater diversity of ecological gradients that can be occupied by species with different niches (Leibold et al., 2004). Yet, lake clusters that showed the highest environmental heterogeneity (e.g., Bolivian‐Brazilian lowlands and Peruvian Andes) did not reveal stronger niche‐based mechanisms relative to dispersal‐based ones (Figure 5). Moreover, the positive relationship between environmental filtering and habitat heterogeneity might depend on spatial extent, because dispersal processes (i.e., dispersal limitation or mass effects) may reduce species sorting at either large or small extents (Zorzal‐Almeida, Soininen, Bini, & Bicudo, 2017). In the Bolivian Andes and Southern Altiplano lake clusters, both spatial and environmental effects correlated with diatom species composition, indicating that diatom metacommunities are niche assembled but constrained either by dispersal limitation or mass effects, or both. While our results are correlative, and thus we cannot unequivocally provide causal evidence for distinguishing between dispersal limitation and mass effects, we associated the significant spatial role to mass effects due to the relatively small spatial extent of these two lake clusters (Supporting Information Appendix S2), according to the expectations of Heino et al. (2015) and Tonkin et al. (2016). In contrast, at the largest spatial extent (e.g., Peruvian Andes), dispersal limitation would be primary in structuring diatom metacommunities.

The structure of lake metacommunities has been often analyzed using exclusively local environmental and spatial effects (Declerck, Winter, Shurin, Suttle, & Matthews, 2013; Heino et al., 2016). Yet climatic effects have not been evaluated as thoroughly, although they may affect environmental filtering (Alahuhta & Heino, 2013; Loewen, 2017). Some latent environmental predictors (e.g., catchment productivity) are a function of climatic and several lake/catchment features (e.g., precipitation, topography) across large scale in tropical regions. For instance, Steinitz‐Kannan, Colinvaux, and Kannan (1983) found that nutrient levels are related to altitude in Andean lakes of Ecuador. Benito et al. (2018) show that geo‐climatic variables might be partially manifested via local limnological variables in Andean lakes of Peru and Ecuador. Here, we used PCA site scores to capture latent environmental variables and likely those local variables (e.g., nutrients) that are spatially patterned and outperformed by macroecological variables in most groups of our study lakes (Benito et al., 2018), and hence use environmental drivers of lakes’ clustering to infer niche‐based assembly processes (e.g., Steinitz‐Kannan et al., 1983). Nonetheless, the relative influence of space over environmental factors, as indicated by variance partitioning (Figure 5), provided limited evidence for niche assembled diatom communities at a regional metacommunity scale. At the biogeographic scale (continental), we found that spatial factors outperformed environmental factors, as well (Benito et al., 2018), suggesting that dispersal‐based processes predominantly control the structuring of lake diatom assemblages in tropical South America at different spatial scales. Rather than being mutually exclusive, species sorting and dispersal dynamics may jointly drive diatom community composition with varying effects mediated by ecological guilds, as discussed below.

Evidence from studies of small spatial scales indicates that diatom guilds can not only track environmental gradients but also spatial factors due to the relationship between life forms and dispersal abilities (Dong et al., 2016; Liu et al., 2013; Riera, Magnuson, Kratz, & Webster, 2000; Vilmi et al., 2017). At continental spatial scales, previous studies demonstrated strong responses of diatom guild distributions in streams to both environmental and spatial factors (Passy, 2017; Soininen et al., 2016). Our results in groups of tropical lakes indicated that high‐profile and low‐profile guilds (“weak dispersers”) were driven by spatial variables along a latitudinal gradient (in terms of uniquely explained variation by geographic component decreasing with latitude; Figure 5). Hence, the high‐profile and low‐profile guilds were less spatially structured in high‐latitude lake clusters, which have more variable climatic conditions (higher seasonality) than equatorial latitudes. Climatic stability with latitude has been suggested to drive a dispersal‐ecological specialization trade‐off at metacommunity level (Zaharescu, Hooda, Burghelea, & Palanca‐Soler, 2016). However, environmental variables can be spatially structured, thereby resulting in shared effects, as revealed in our study (Supporting Information Appendix S6—Table S6.2). These effects may lead to spurious interpretations of spatial effects as proxies of dispersal dynamics. Nonetheless, the total shared effect among environment, geographic, and topographic components also showed a clear decreasing latitudinal pattern (Figure 5). This suggests that prevalence of ecological guild variation in response to higher regionally structured environment toward the equator might explain the formation of the latitudinal diatom metacommunity gradient.

The detection of significant environmental effects on the motile guild supported the importance of niche‐based processes in affecting loosely attached diatoms (“strong dispersers”) (e.g. Jocque, Field, Brendonck, & Meester, 2010; Vilmi et al., 2017). This is particularly evident in the Bolivian‐Brazilian lowlands, where the strength of the environmental component outperformed the effect of spatial factors on diatom community composition, likely due to the high environmental heterogeneity of tropical floodplain lakes, which is evident in the Pantanal lakes (McGlue et al., 2011). These hydrologically pulsing environments favor the dispersal of aquatic microorganisms among habitats (Cardoso et al., 2017; Devercelli, Scarabotti, Mayora, Schneider, & Giri, 2016; Dias et al., 2016). Although local environmental variables appeared to exert a major control on planktic communities (Supporting Information Appendix S5—Figure S5.3), variance partitioning did not provide support for niche‐based assembly processes for the planktic guild among‐lake clusters, in which pure spatial controls were mostly detected. Dispersal‐related mechanisms in diatom metacommunities have also been suggested in mountainous areas where constrained aerial dispersed is likely due to high elevational gradients and step valleys (Dong et al., 2016; Jamoneau, Passy, Soininen, Leboucher, & Tison‐Rosebery, 2018). Nonetheless, we cannot exclude the possibility that the nonsignificant environmental structure at metacommunity level is a product of the low abundance of planktic taxa in our database (10% of the total taxa), or the noninclusion of ecologically important processes for phytoplankton communities (e.g., biotic interactions, trophic state) (Nabout, Siqueira, Bini, & de Nogueira, 2009).

Landscape features mediate the importance of regional processes in shaping metacommunities by either promoting or limiting dispersal (Badgley et al., 2017). In our study, a significant proportion of community composition in all diatom guilds was explained by geographic distances. Similar results have been found by Zorzal‐Almeida et al. (2017) in a set of tropical reservoirs. However, topographic constraints, such as mountain barriers, may yield more ecologically informative relationships among sites than straight‐line distances (Dong et al., 2016). Topographic distances had only a significant relationship with diatom guilds loosely attached to the substrate (motile and planktic) in certain lake clusters (Bolivian‐Brazilian lowlands and Peruvian Andes). These two regions mostly correspond to lake clusters characterized by a combination of rugged topography and relatively low % aquatic habitat (Supporting Information Appendix S2), suggesting that complex topography in isolated lake systems indeed exerts constraints for motile and planktic guild distribution. Unexpectedly, in the topographically complex lake clusters of the Andes and lowlands of Ecuador and Colombia, spatial effects due to rugged topography did not appear to exert influence on the community composition of these two diatom guilds, as indicated by the stronger influence of geographical distance. Subtle differences in topographic heterogeneity and hydrological connections between lakes through riverine network (not accounted for in this study) might explain the varied spatial control in diatom guilds. These differences may further contribute to the development of regionally distinct diatom metacommunities in tropical South America.

Our results indicating shifts in ecological drivers on diatom metacommunity structuring across climatic and physiographically distinct lake regions would not have been detected without a functional approach. These findings are in accord with those of Vilmi et al. (2017) in lakes and Jamoneau et al. (2018) in streams, who found different responses to environmental and spatial factors when diatom ecological guilds are analyzed individually, rather than entire assemblages. Nonetheless, it is hard to conclude that large‐scale ecological patterns can be explained by environmental and/or spatial variables, given the complex spatial‐temporal mechanisms of community assembly for a certain metacommunity (Brown et al., 2017; Padial et al., 2014). For the present study, two independent lines of evidence supported this statement. First, percentages of explained variation among models for the whole diatom community and for each ecological guild separately were low (adjusted *R* ^2^ = 1%–20%). Among others, this can be attributed to intraguild interactions that alter fundamental ecological niches via competition‐facilitation effects (Passy, 2017), the noninclusion of explicit measures of nutrients, which are a major driver of diatom community composition over large‐scale surveys (Soininen et al., 2016; Verleyen et al., 2009; Vyverman et al., 2007; Winegardner et al., 2015), and methodological difficulties in measuring multiple turnovers in community composition (Heino et al., 2016). Although such low values are comparable with other large‐scale studies that analyze aquatic metacommunities (adjusted *R* ^2^ = 1%–35%) (Bie et al., 2012; Hájek et al., 2011; Heino et al., 2016; Padial et al., 2014; Pandit, Kolasa, & Cottenie, 2009; Soininen et al., 2016; Zorzal‐Almeida et al., 2017), our study indicates that tropical lake diatom metacommunities are highly dynamic and that any inference from one‐off snapshot sampling may be misleading. This is shown by significant temporal effects found in the diatom data from the analyses of sediment surface, periphyton, and plankton communities, as well as the entire diatom species matrix. Second, the effects of environmental and spatial variables were not significant in the Southern Altiplano metacommunity (Figure 5). Previous studies in lentic systems suggest that null results can result from unmeasured fine‐scale environmental variability (Nabout et al., 2009). This fact, coupled with the extreme environmental conditions found in the shallow hypersaline lakes (“salares”) of the Southern Altiplano (Sylvestre et al., 2001), could further explain these null results.

Our study revealed broadly similar patterns to those that were generated from the analyses of diatom communities when considering all the species versus separating species from sediment surface and periphyton habitats. This suggests that the rich benthic species pool closely tracks different environmental and spatial influences on lacustrine community composition, and thus reinforces the guild‐specific metacommunity model to account for biogeographic variation (i.e., latitudinal gradient). Combining two disciplines at disparate spatial scales (metacommunity and biogeography) might help guide future diatom research, for example, in exploring the evolutionary origin of the marked interhemispheric differences in species composition in response to past tectonic and climatic events and their role in the strong latitudinal gradient of diatom diversity (Verleyen et al., 2009).

## CONFLICT OF INTEREST

None declared.

## AUTHOR CONTRIBUTIONS

XB and SF conceived the ideas; XB, SF, MK, MV, and MM analyzed and processed individual diatom data sets; XB analyzed the data and lead the writing of the manuscript with contributions of all the authors.

## DATA ACCESSIBILITY

Diatom community data, water chemistry, and geo‐climatic variables of lakes are available at Dryad Digital Repository http://doi.org/doi:10.5061/dryad.6jk8h77.
