# Supplementary material for: Lake regionalization and diatom metacommunity structuring in tropical South America
Source: Ecol Evol. 2018 Jul 13;8(16):7865–78. doi: 10.1002/ece3.4305 (PMC6145031; doi:10.1002/ece3.4305)
Supplement: Supplementary file 1 [file ECE3-8-7865-s001.docx]

**SUPORTING INFORMATION**

**Lake regionalization and diatom metacommunity structuring in tropical South America**

Xavier Benito, Sherilyn C. Fritz, Miriam Steinitz-Kannan, Maria I. Velez, Michael M. McGlue

**Appendix S1. Sampling site information**

**Table S1.1** List of study lakes (n = 195), with the sample type, number of samples (total = 303), year of sampling, and geographical coordinates. Regions and lakes are arranged by latitude (from lower to higher). Latitude and longitude are represented in decimal degrees.

| **Region** | **Lake** | **Sample type** | **N samples** | **Year of sampling** | **Latitude** | **Longitude** |
| --- | --- | --- | --- | --- | --- | --- |
| **Colombia lowlands** |  |  |  |  |  |  |
| N=6 | Ayapel | sediment surface and plankton | 4 | 2003 | 8.32 | -75.10 |
|  | Antioquia | periphyton and plankton | 9 | 2003 | 6.57 | -75.80 |
|  | Juncal | periphyton and plankton | 3 | 2003 | 5.13 | -72.75 |
|  | Carimagua | periphyton and plankton | 5 | 2003 | 4.58 | -71.33 |
|  | Pinal | sediment surface and plankton | 3 | 2003 | 4.13 | -73.38 |
|  | Margaritas | sediment surface and plankton | 3 | 2003 | 3.38 | -73.42 |
| **Colombia Andes** |  |  |  |  |  |  |
| N=9 | Belmira | sediment surface and plankton | 3 | 2001 | 6.62 | -75.64 |
|  | Guarne | sediment surface and plankton | 2 | 2001 | 6.27 | -75.52 |
|  | Fuquene | sediment surface | 4 | 2003 | 5.47 | -73.75 |
|  | Cucunoba | sediment surface and plankton | 2 | 2003 | 5.28 | -73.78 |
|  | Suesca | sediment surface and plankton | 2 | 2003 | 5.18 | -73.78 |
|  | Guatavita | sediment surface and plankton | 2 | 2003 | 4.98 | -73.78 |
|  | Herrera | periphyton and plankton | 2 | 2003 | 4.69 | -74.27 |
|  | Verjon | periphyton and plankton | 3 | 2003 | 4.56 | -74.02 |
|  | Ubaque | plankton | 12 | 2014 | 4.5 | -73.93 |
| **Ecuador Amazon** |  |  |  |  |  |  |
| N=17 | Caimancocha | sediment surface and plankton | 1 | 1977-1981 | 0.01 | -76.21 |
|  | Macurococha | sediment surface and plankton | 1 | 1977-1981 | 0.00 | -76.21 |
|  | Cuyabeno | sediment surface and plankton | 1 | 1977-1981 | -0.01 | -76.18 |
|  | Mateococha | sediment surface and plankton | 1 | 1977-1981 | -0.01 | -76.22 |
|  | Santa Cecilia | sediment surface and plankton | 1 | 1977-1981 | -0.07 | -77.02 |
|  | Texaco Pond | sediment surface and plankton | 1 | 1977-1981 | -0.08 | -76.88 |
|  | L. Agrio | sediment surface and plankton | 1 | 1977-1981 | -0.12 | -76.92 |
|  | Limoncocha | sediment surface and plankton | 1 | 1977-1981 | -0.40 | -76.63 |
|  | Mandicocha | sediment surface and plankton | 1 | 1977-1981 | -0.47 | -76.46 |
|  | Taracoa | sediment surface and plankton | 1 | 1977-1981 | -0.55 | -76.75 |
|  | Garzacocha | sediment surface and plankton | 1 | 1977-1981 | -0.58 | -76.33 |
|  | Zancudococha | sediment surface and plankton | 1 | 1977-1981 | -0.60 | -75.48 |
|  | Añangucocha | sediment surface and plankton | 1 | 1977-1981 | -0.67 | -76.42 |
|  | Pecari | sediment surface and plankton | 1 | 1977-1981 | -1.53 | -78.00 |
|  | Puyo | sediment surface and plankton | 1 | 1977-1981 | -1.55 | -78.00 |
|  | Ayauchi | sediment surface and plankton | 1 | 1977-1981 | -2.08 | -78.02 |
|  | L. Kumpak | sediment surface and plankton | 1 | 1977-1981 | -3.03 | -77.82 |
| **Ecuador-Interandean** |  | sediment surface and plankton |  | 1977-1981 |  |  |
| N=11 | Yahuarcocha | sediment surface and plankton | 1 | 1977-1981 | 0.38 | -78.08 |
|  | Guangopolo | sediment surface and plankton | 1 | 1977-1981 | 0.25 | -78.48 |
|  | Cunro | sediment surface and plankton | 1 | 1977-1981 | 0.24 | -78.09 |
|  | San Pablo | sediment surface and plankton | 1 | 1977-1981 | 0.22 | -78.23 |
|  | Zuleta | sediment surface and plankton | 1 | 1977-1981 | 0.20 | -78.10 |
|  | Yambo | sediment surface and plankton | 1 | 1977-1981 | -1.12 | -78.58 |
|  | La Moya | sediment surface and plankton | 1 | 1977-1981 | -1.33 | -78.52 |
|  | Rumtum | sediment surface and plankton | 1 | 1977-1981 | -1.45 | -78.73 |
|  | San Antonio | sediment surface and plankton | 1 | 1977-1981 | -1.62 | -78.63 |
|  | Colta | sediment surface and plankton | 1 | 1977-1981 | -1.75 | -78.73 |
|  | Uzhupud | sediment surface and plankton | 1 | 1977-1981 | -2.83 | -78.80 |
| **Ecuador Andes** |  | sediment surface and plankton |  | 1977-1981 |  |  |
| N=22 | Cuicocha | sediment surface and plankton | 1 | 1977-1981 | 0.30 | -78.37 |
|  | Caricoha | sediment surface and plankton | 1 | 1977-1981 | 0.14 | -78.27 |
|  | Yanacocha | sediment surface and plankton | 1 | 1977-1981 | 0.13 | -78.25 |
|  | San Marcos | sediment surface and plankton | 1 | 1977-1981 | 0.12 | -78.42 |
|  | Huarmicocha | sediment surface and plankton | 1 | 1977-1981 | 0.11 | -78.27 |
|  | Limpiopungo | sediment surface and plankton | 1 | 1977-1981 | -0.25 | -78.50 |
|  | Papallacta | sediment surface and plankton | 1 | 1977-1981 | -0.37 | -78.17 |
|  | La Ciénega | sediment surface and plankton | 1 | 1977-1981 | -0.77 | -78.62 |
|  | Quilotoa | sediment surface and plankton | 1 | 1977-1981 | -0.86 | -78.90 |
|  | Pisayambo | sediment surface and plankton | 1 | 1977-1981 | -1.08 | -78.39 |
|  | Anteojos | sediment surface and plankton | 1 | 1977-1981 | -1.10 | -78.34 |
|  | Cochabamba | sediment surface and plankton | 1 | 1977-1981 | -1.50 | -78.72 |
|  | Tambo | sediment surface and plankton | 1 | 1977-1981 | -1.83 | -78.37 |
|  | Culebrillas | sediment surface and plankton | 1 | 1977-1981 | -2.42 | -78.86 |
|  | Fondococha | sediment surface | 1 | 2011 | -2.76 | -79.24 |
|  | Chorreras | sediment surface | 1 | 2011 | -2.77 | -79.16 |
|  | Patoquinoas | sediment surface | 1 | 2011 | -2.78 | -79.21 |
|  | Illincocha | sediment surface and plankton | 1 | 1977-1981 | -2.79 | -79.22 |
|  | Toreadora | sediment surface | 1 | 2011 | -2.81 | -79.24 |
|  | Llaviucu | sediment surface | 1 | 2011 | -2.84 | -79.15 |
|  | Lagunas de Napalé | sediment surface | 1 | 2011 | -2.89 | -79.30 |
|  | Cañar pond | sediment surface and plankton | 1 | 1977-1981 | -4.62 | -79.42 |
| **Junin Plain** |  |  |  |  |  |  |
| N=31 | L. Tauli, shore | sediment surface | 2 | 1999 | -10.72 | -76.07 |
|  | L. Ccochachuycho, shore | sediment surface | 1 | 1999 | -10.76 | -76.07 |
|  | L. Lulicocha, shore | sediment surface | 2 | 1999 | -10.78 | -76.10 |
|  | Junin 10, Tambo del Sol | sediment surface | 2 | 1999 | -10.81 | -76.13 |
|  | Junin 9, ditch before Shelby | sediment surface | 2 | 1999 | -10.81 | -76.23 |
|  | L. Yanacocha, shore | sediment surface | 2 | 1999 | -10.83 | -76.04 |
|  | L. Purun, center | sediment surface | 1 | 1999 | -10.84 | -76.46 |
|  | L. Purun, shore | periphyton | 2 | 1999 | -10.84 | -76.45 |
|  | Yanacocha 3, spring | periphyton | 2 | 1999 | -10.86 | -75.97 |
|  | Yanacocha 1, wetland | periphyton | 1 | 1999 | -10.86 | -75.99 |
|  | Yanacocha 2 | periphyton | 1 | 1999 | -10.86 | -75.98 |
|  | Yanacocha 4, outlet channel | periphyton | 1 | 1999 | -10.87 | -76.00 |
|  | Junin 14, Palcamayo R. | periphyton | 1 | 1999 | -10.91 | -76.05 |
|  | Junin 8, Upamayo Bridge | periphyton | 2 | 1999 | -10.92 | -76.26 |
|  | Junin 11, Gunoc | sediment surface | 3 | 1999 | -10.94 | -76.05 |
|  | Junin 7, San Pedro de Pari | periphyton | 2 | 1999 | -10.96 | -76.23 |
|  | Junin 13, Qda. Anascanchi | periphyton | 2 | 1999 | -10.98 | -76.04 |
|  | L. Junin, center | sediment surface | 2 | 1999 | -11.00 | -76.06 |
|  | Junin 12, Chacpas | periphyton | 1 | 1999 | -11.00 | -76.01 |
|  | Junin 6, Chuchucancha | sediment surface | 2 | 1999 | -11.04 | -76.19 |
|  | Junin 5, Conoc spring | sediment surface | 2 | 1999 | -11.06 | -76.16 |
|  | L. Junin, shore | periphyton | 2 | 1999 | -11.06 | -76.16 |
|  | Junin 4, Ondores spring | periphyton | 2 | 1999 | -11.08 | -76.15 |
|  | L. Alcacocha, shore | periphyton | 1 | 1999 | -11.08 | -75.94 |
|  | Junin 2, Warmipuquio | periphyton | 3 | 1999 | -11.11 | -76.09 |
|  | Junin 3, spring 2 | periphyton | 1 | 1999 | -11.12 | -76.09 |
|  | Junin 1, Chacachimpa | periphyton | 1 | 1999 | -11.17 | -76.02 |
|  | Huagapo Cave, inside | periphyton | 1 | 1999 | -11.27 | -75.79 |
|  | Huagapo Cave, outside | periphyton | 1 | 1999 | -11.27 | -75.79 |
|  | L. Paca | periphyton | 2 | 1999 | -11.72 | -75.51 |
|  | L. Paca, 12m deep | sediment surface | 1 | 1999 | -11.73 | -75.51 |
| **Amazon Lowlands (Perú-Bolivia)** |  |  |  |  |  |  |
| N=8 | Cocha Maizal | sediment surface | 1 | 2013 | -11.84 | -71.47 |
|  | Cocha Nueva | sediment surface | 1 | 2013 | -11.86 | -71.46 |
|  | Cocha Totora | sediment surface | 1 | 2013 | -11.88 | -71.39 |
|  | Cocha Cashu | sediment surface | 1 | 2013 | -11.88 | -71.41 |
|  | Cocha Gallareta | sediment surface | 1 | 2013 | -11.95 | -71.32 |
|  | Cocha Salvador | sediment surface | 1 | 2013 | -11.99 | -71.22 |
|  | Laguna Chalalan | sediment surface | 1 | 2012 | -14.43 | -67.92 |
|  | Laguna Santa Rosa | sediment surface | 1 | 2012 | -14.49 | -67.87 |
| **Perú Andes (Eastern Cordillera)** |  |  |  |  |  |  |
| N=4 | Huamanmarca | sediment surface | 1 | 2009 | -13.03 | -72.37 |
|  | Miski | sediment surface | 1 | 2009 | -13.03 | -72.37 |
|  | Pacucha | sediment surface | 1 | 2009 | -13.62 | -73.32 |
|  | Acopia | sediment surface | 1 | 2009 | -14.08 | -71.52 |
| **Cusco** |  |  |  |  |  |  |
| N=17 | Cusco-PLS-2 | sediment surface | 1 | 2011 | -13.40 | -72.13 |
|  | Cusco-PLS-13 | sediment surface | 1 | 2011 | -13.40 | -71.69 |
|  | Cusco-PLS-1 | sediment surface | 1 | 2011 | -13.42 | -72.05 |
|  | Cusco-PLS-11 | sediment surface | 1 | 2011 | -13.62 | -71.72 |
|  | Cusco-PLS-5 | sediment surface | 1 | 2011 | -13.86 | -70.25 |
|  | Cusco-PLS-7 | sediment surface | 1 | 2011 | -13.86 | -70.31 |
|  | Cusco-PLS-8 | sediment surface | 1 | 2011 | -13.91 | -70.30 |
|  | Cusco-CH1 | sediment surface | 1 | 2011 | -13.92 | -70.87 |
|  | Cusco-PLS-6 | sediment surface | 1 | 2011 | -13.93 | -70.31 |
|  | Cusco-DP1 | sediment surface | 1 | 2011 | -13.94 | -70.90 |
|  | Cusco-YC1 | sediment surface | 1 | 2011 | -13.95 | -70.87 |
|  | Cusco-IA1 | sediment surface | 1 | 2011 | -13.95 | -70.90 |
|  | Cusco-PLS-12 | sediment surface | 1 | 2011 | -14.13 | -71.46 |
|  | Cusco-PLS-3 | sediment surface | 1 | 2011 | -14.58 | -71.72 |
|  | Cusco-PLS-4 | sediment surface | 1 | 2011 | -14.59 | -71.71 |
|  | Cusco-PLS-9 | sediment surface | 1 | 2011 | -14.73 | -70.50 |
|  | Cusco-PLS-10 | sediment surface | 1 | 2011 | -15.09 | -70.94 |
| **Beni lowlands** |  |  |  |  |  |  |
| N=6 | Lagoon Huachi | sediment surface | 1 | 2006 | -14.30 | -63.40 |
|  | Lagoon Belen | sediment surface | 1 | 2006 | -14.45 | -64.85 |
|  | Lagoon Coitarama | sediment surface | 1 | 2006 | -14.50 | -64.87 |
|  | Lagoon Cernandez | sediment surface | 1 | 2006 | -14.69 | -64.77 |
|  | Lagoon Puente de Ibaré | sediment surface | 1 | 2006 | -14.87 | -64.97 |
|  | Lagoon Azul | sediment surface | 1 | 2006 | -14.99 | -64.81 |
| **Titicaca** |  |  |  |  |  |  |
| N=6 | Suches | periphyton |  | 1998 | -15.66 | -69.13 |
|  | Titicaca Lake | sediment surface and plankton | 23 | 1996-1998 | -15.90 | -69.43 |
|  | Isla del Sol | periphyton | 1 | 1998 | -16.00 | -69.18 |
|  | Ilave | periphyton | 3 | 1998 | -16.09 | -69.63 |
|  | Huatajata | periphyton | 3 | 1998 | -16.21 | -68.69 |
|  | Yunguyo Bay | sediment surface and plankton | 9 | 1992 | -16.22 | -69.13 |
| **Perú Andes (Puno)** |  |  |  |  |  |  |
| N=19 | Chacas | sediment surface | 1 | 2003 | -15.41 | -70.20 |
|  | Umapata | sediment surface | 1 | 2003 | -15.54 | -70.05 |
|  | Pacuna | sediment surface | 1 | 2003 | -15.58 | -70.21 |
|  | Lake 6 | sediment surface | 1 | 2003 | -15.66 | -70.20 |
|  | Lagunillas | sediment surface | 1 | 2003 | -15.75 | -70.68 |
|  | Sara Cocha | sediment surface | 1 | 2003 | -15.78 | -70.61 |
|  | Ululumasa | sediment surface | 1 | 2003 | -15.78 | -70.60 |
|  | Maquera | sediment surface | 1 | 2003 | -15.88 | -70.56 |
|  | Calzada | sediment surface | 1 | 2003 | -15.90 | -70.51 |
|  | Khara Kkota (north) | sediment surface | 1 | 2003 | -16.13 | -68.36 |
|  | Estrellani | sediment surface | 1 | 2003 | -16.34 | -68.04 |
|  | Jacumarini | sediment surface | 1 | 2003 | -16.36 | -70.39 |
|  | Asiruni | sediment surface | 1 | 2003 | -16.39 | -70.37 |
|  | Laja | sediment surface | 1 | 2003 | -16.53 | -68.39 |
|  | Jachcha Kkota | sediment surface | 1 | 2003 | -16.58 | -68.17 |
|  | Chara Nkkota | sediment surface | 1 | 2003 | -16.58 | -68.16 |
|  | Lake 27 | sediment surface | 1 | 2003 | -16.62 | -68.27 |
|  | Lake 23 | sediment surface | 1 | 2003 | -16.68 | -68.32 |
|  | Lake 34 | sediment surface | 1 | 2003 | -16.91 | -68.95 |
| **Cochabamba** |  |  |  |  |  |  |
| N=1 | Laguna Challacaba | sediment surface | 1 | 2007 | -17.560 | -65.57 |
| **Desaguadero** |  |  |  |  |  |  |
| N=20 | Rio Desaguadero: downstream from Desaguadero | periphyton | 1 | 2001 | -16.64 | -68.97 |
|  | Rio Desaguadero: Nazacara N. | sediment surface | 1 | 2001 | -16.88 | -68.80 |
|  | Laguna Desaguadero | sediment surface | 1 | 2001 | -16.98 | -68.79 |
|  | Rio Desaguadero at Parco Khota | periphyton | 1 | 2001 | -17.21 | -68.68 |
|  | Rio Mauri | periphyton | 1 | 2001 | -17.29 | -68.63 |
|  | Rio Desaguadero Bridge (pipeline) | periphyton | 1 | 2001 | -17.31 | -68.62 |
|  | Flood Plain Pond near Pipeline | sediment surface | 1 | 2001 | -17.33 | -68.62 |
|  | Laguna Blanca S. Rio Jalsuri Uma | sediment surface | 1 | 2001 | -17.47 | -68.55 |
|  | Salcro Tarquiamaya | periphyton | 1 | 2001 | -17.48 | -68.59 |
|  | Ibirizu River | periphyton | 1 | 2001 | -17.50 | -65.27 |
|  | Rio Quebrada Huajra Uma | periphyton | 1 | 2001 | -17.50 | -68.42 |
|  | Huancaroma Dairy | sediment surface | 1 | 2001 | -17.66 | -67.48 |
|  | Laguna Soledad (Uru Uru) | sediment surface | 1 | 2001 | -17.75 | -67.31 |
|  | Rio Desaguadero Balsa Crossing | sediment surface | 1 | 2001 | -18.20 | -67.09 |
|  | Rio Desaguadero | periphyton | 1 | 2001 | -18.35 | -67.04 |
|  | Rio Desaguadero: Poopo (town) | periphyton | 1 | 2001 | -18.37 | -67.05 |
|  | Rio Poopo drainage | periphyton | 1 | 2001 | -18.38 | -67.02 |
|  | Road tracks & N Lago Poopo plain | sediment surface | 1 | 2001 | -18.55 | -67.00 |
|  | N Lago Poopo plain | sediment surface | 1 | 2001 | -18.57 | -66.95 |
|  | Laguna Chairiri | sediment surface | 1 | 2001 | -22.53 | -67.65 |
| **Pantanal** |  |  |  |  |  |  |
| N=3 | Lagoon Gaiba | sediment surface | 1 | 2001 | -17.78 | -57.72 |
|  | Lagoon Mandiore | sediment surface | 1 | 2001 | -18.10 | -57.55 |
|  | Baia Vermelha | sediment surface | 1 | 2011 | -18.39 | -57.51 |
| **Sud Lipez** |  |  |  |  |  |  |
| N=14 | Salar de Uyuni | periphyton | 1 | 1991 | -20.14 | -67.66 |
|  | Canapa | periphyton | 1 | 1991 | -21.00 | -68.02 |
|  | Chulluncani | sediment surface | 2 | 2002 | -21.53 | -67.88 |
|  | Hedionda | sediment surface | 1 | 2002 | -21.57 | -68.07 |
|  | Chiar Kkota | sediment surface | 1 | 2002 | -21.58 | -68.07 |
|  | Honda | sediment surface | 1 | 2002 | -21.62 | -68.07 |
|  | Pujio | sediment surface | 1 | 2002 | -21.62 | -68.07 |
|  | Ballivian | sediment surface | 1 | 2002 | -21.63 | -68.08 |
|  | Ramaditas | sediment surface | 1 | 2002 | -21.64 | -68.08 |
|  | Pastos Grandes | sediment surface | 10 | 2002 | -21.65 | -67.78 |
|  | Laguna Cachi | sediment surface | 1 | 2002 | -21.73 | -67.95 |
|  | Laguna Colorada | sediment surface | 2 | 2002 | -21.77 | -67.72 |
|  | Puripica | sediment surface | 1 | 2002 | -22.52 | -67.50 |
|  | Laguna Verde | sediment surface | 1 | 2002 | -22.80 | -67.80 |
| **Mar Chiquita** |  |  |  |  |  |  |
| N=1 | Mar Chiquita | sediment surface | 1 | 2008 | -30.83 | -62.57 |

**Table S1.2** Mean and range (between brackets) of the analyzed limnological and geo-climatic data for each study region; n correspond to the number of the analyzed samples (n = 303), with %NA showing the proportion of missing values. Regions are arranged from low to high latitude. MAT=Mean Annual Temperature; MAP=Mean Annual Precipitation. P = precipitation; T = temperature; TRI = Terrain Ruggedness Index.

| **Region** | | **Temperature** | **pH** | **Conductivity** | **Latitude** | **Longitude** | **MAT** | **T seasonality** | **MAP** | **P seasonality** | **Elevation** | **TRI** | **% aquatic habitat** | **Connectivity** | **Area lake** |
| --- | --- | --- | --- | --- | --- | --- | --- | --- | --- | --- | --- | --- | --- | --- | --- |
|  | n | deg C |  | µS/cm | Decimal  degree | Decimal  degree | deg C | mm | mm | Coefficient variation (mm) | m |  | % freshwater in 50 km^2^ | N water bodies in 50 km^2^ | km^2^ |
| Colombia lowlands | 27 | 28.74 | 5.45 | 23.55 | 5.67 | -74.00 | 26.28 | 656.30 | 2316.63 | 54.81 | 316.96 | 4.19 | 6.86 | 0.01 | 11.93 |
|  |  | (28.74-28.74) | (4.52-6.87) | (7.6-59.25) | (3.38-8.32) | (-75.80--71.33) | (24.1-27.9) | (469-858) | (1264-4116) | (47-65) | (31-648) | (0-11.875) | (0-22.357) | (0-0.021) | (0.02-46) |
|  | *%NA* | *32* | *32* | *32* |  |  |  |  |  |  |  |  |  |  |  |
| Colombia Andes | 32 | 17.13 | 6.90 | 323.22 | 5.07 | -74.17 | 14.10 | 357.38 | 1316.88 | 40.78 | 2377.66 | 6.07 | 0.97 | 0.00 | 6.15 |
|  |  | (13-19) | (6.08-9.47) | (8.4-1220) | (4.5-6.62) | (-75.64--73.752) | (8.2-16.0) | (202-403) | (627-3009) | (30-50) | (1655-3397) | (0-27.75) | (0.047-4.413) | (0-0.004) | (0.01-35.27) |
|  | *%NA* | *46* | *46* | *46* |  |  |  |  |  |  |  |  |  |  |  |
| Ecuador-Amazonia | 17 | 26.71 | 6.60 | 114.76 | -0.69 | -76.80 | 24.42 | 459.18 | 3326.71 | 17.06 | 510.12 | 3.11 | 17.24 | 0.01 | 808.65 |
|  |  | (23.45-28.7) | (4.81-9) | (11.7-373) | (-3.03-0.00) | (-78.01--75.48) | (21.1-25.9) | (400-599) | (2357-4379) | (13-25) | (224-1630) | (0-10.75) | (2.327-54.938) | (0.001-0.01) | (0-5541.83) |
|  | *%NA* | *32* | *15* | *15* |  |  |  |  |  |  |  |  |  |  |  |
| Ecuador-Interandean | 12 | 17.13 | 7.63 | 703.21 | -0.71 | -78.45 | 12.82 | 367.00 | 852.75 | 35.83 | 2928.42 | 14.14 | 0.31 | 0.00 | 27.93 |
|  |  | (14.03-21.7) | (6.7-8.75) | (163.3-1950) | (-2.833-0.38) | (-78.80--78.08) | (5.8-166) | (138-668) | (533-1099) | (21-49) | (2133-3728) | (0-77.125) | (0-0.674) | (0-0.002) | (0.03-141.2) |
|  | *%NA* | *13* | *13* | *9* |  |  |  |  |  |  |  |  |  |  |  |
| Ecuador Andes | 23 | 10.95 | 7.16 | 1037.61 | -1.72 | -78.80 | 7.91 | 420.61 | 1037.00 | 32.04 | 3545.35 | 5.25 | 0.20 | 0.00 | 8.81 |
|  |  | (5-17) | (5-8.6) | (10-15600) | (-4.61-0.13) | (-79.41--78.16) | (4.7-16.9) | (193-718) | (671-1353) | (17-77) | (1910-4093) | (0-34.375) | (0-0.674) | (0-0.002) | (0-190.1) |
|  | *%NA* | *17* | *17* | *23* |  |  |  |  |  |  |  |  |  |  |  |
| JuninPlain | 49 | 12.39 | 8.09 | 384.95 | -11.00 | -76.07 | 6.03 | 770.41 | 1003.29 | 60.06 | 3970.63 | 4.72 | 13.45 | 0.00 | 150.87 |
|  |  | (9.4-16.8) | (6.75-9.13) | (72.5-1184) | (-11.72--10.72) | (-76.45--75.50) | (4.0-11.5) | (693-840) | (673-1260) | (55-74) | (3582-4233) | (0-27.75) | (0.047-16.322) | (0-0.004) | (0-396.47) |
|  | *%NA* | *4* | *4* | *8* |  |  |  |  |  |  |  |  |  |  |  |
| Amazon Lowlands (Perú-Bolivia) | 8 | 29.16 | 7.73 | 167.18 | -12.54 | -70.51 | 25.04 | 875.00 | 2337.63 | 45.75 | 406.00 | 3.42 | 1.26 | 0.01 | 1.45 |
|  |  | (24.9-32.7) | (7.16-8.62) | (12.4-317) | (-14.49--11.84) | (-71.46--67.86) | (24.6-25.2) | (670-1454) | (1892-2612) | (44-49) | (331-509) | (0-7.875) | (0.2-1.927) | (0-0.008) | (0.43-4.58) |
|  | *%NA* | *-* | *-* | *-* |  |  |  |  |  |  |  |  |  |  |  |
| Perú Andes (Eastern Cordillera) | 4 | 14.00 | 8.46 | 834.00 | -13.44 | -72.40 | 9.50 | 1277.75 | 822.75 | 77.50 | 3435.75 | 7.63 | 0.83 | 0.00 | 96.50 |
|  |  | (14-14) | (8.46-8.46) | (345-1323) | (-14.08--13.03) | (-73.31--71.52) | (7.5-13.1) | (969-1757) | (778-937) | (74-86) | (3201-4020) | (0-19.625) | (0.247-2.512) | (0-0.001) | (52.4-190.1) |
|  | *%NA* | *25* | *25* | *25* |  |  |  |  |  |  |  |  |  |  |  |
| Cusco | 17 | 10.99 | 8.13 | 437.00 | -14.02 | -71.10 | 5.51 | 1784.00 | 755.29 | 81.59 | 4238.00 | 2.83 | 0.26 | 0.00 | 1.50 |
|  |  | (4.6-17.3) | (7.52-9.74) | (5.6-3205) | (-15.08--13.39) | (-72.12--70.25) | (0.8-12.6) | (1208-2327) | (569-821) | (71-94) | (3083-5070) | (0-8.375) | (0.009-2.512) | (0-0.002) | (0.01-9) |
|  | *%NA* | *-* | *-* | *-* |  |  |  |  |  |  |  |  |  |  |  |
| Beni lowlands | 6 | 28.59 | 7.50 | 46.25 | -14.63 | -64.61 | 257.17 | 1485.33 | 1784.17 | 5.97 | 168.17 | 1.38 | 7.46 | 0.00 | 14.29 |
|  |  | (22.6-33) | (6.68-8.74) | (25-133) | (-14.98--14.30) | (-64.97--63.40) | (255-258) | (1262-1623) | (1434-1973) | (5.7-6.5) | (155-223) | (0-3.375) | (4.159-9.511) | (0.002-0.006) | (0.11-58.86) |
|  | *%NA* | *-* | *-* | *-* |  |  |  |  |  |  |  |  |  |  |  |
| Titicaca | 36 | 13.00 | 8.30 | 1380.00 | -15.93 | -69.37 | 89.33 | 1378.54 | 866.38 | 8.24 | 3845.71 | 1.04 | 57.59 | 0.00 | 6674.08 |
|  |  | (13-13) | (8.3-8.3) | (1380-1380) | (-16.24--15.25) | (-69.86--68.68) | (46-101) | (1196-1941) | (659-1113) | (7.3-8.7) | (3823-4374) | (0-16.625) | (0.024-88.174) | (0-0.003) | (52.4-7489.27) |
|  | *%NA* | *-* | *-* | *-* |  |  |  |  |  |  |  |  |  |  |  |
| Perú Andes (Puno) | 19 | 12.53 | 8.82 | 478.55 | -16.13 | -69.53 | 65.26 | 1818.05 | 628.58 | 8.95 | 4161.95 | 3.36 | 1.66 | 0.00 | 4.24 |
|  |  | (6.77-22.07) | (7.29-10.28) | (19.72-1910) | (-16.91--15.40) | (-70.67--68.04) | (23-95) | (1525-2407) | (541-728) | (7.6-10.5) | (3744-4626) | (0-34.75) | (0.007-6.201) | (0-0.006) | (0-50.16) |
|  | *%NA* | *-* | *-* | *-* |  |  |  |  |  |  |  |  |  |  |  |
| Cochabamba | 1 | NA | 9.45 | 332.00 | -17.60 | -65.57 | 123.00 | 2124.00 | 467.00 | 10.50 | 3628.00 | 0.00 | 0.87 | 0.00 | 122.10 |
|  |  |  | (9.45-9.45) | (332-332) | (-17.59--17.59) | (-65.56--65.56) | (123-123) | (2124-2124) | (467-467) | (10.5-10.5) | (3628-3628) | (0-0) | (0.871-0.871) | (0.002-0.002) | (122.1-122.1) |
|  | *%NA* | *100* | *-* | *-* |  |  |  |  |  |  |  |  |  |  |  |
| Desaguadero | 20 | NA | 6.09 | 39388.70 | -17.91 | -67.94 | 85.75 | 2644.95 | 378.75 | 10.50 | 3752.35 | 2.44 | 5.41 | 0.01 | 72.95 |
|  |  |  | (4.5-7.5) | (258-351000) | (-22.52--16.64) | (-68.96--66.95) | (45-97) | (2059-3357) | (50-636) | (9.3-14.8) | (3628-4357) | (0.25-9.375) | (0.134-20.355) | (0.002-0.014) | (4.86-183.9) |
|  | *%NA* | *100* | *12* | *8* |  |  |  |  |  |  |  |  |  |  |  |
| Pantanal | 3 | NA | 7.90 | 865.02 | -18.09 | -57.59 | 259.67 | 2087.33 | 1169.67 | 6.00 | 152.00 | 0.00 | 45.89 | 0.01 | 129.03 |
|  |  |  | (7.9-7.9) | (0.04-1730) | (-18.39--17.78) | (-57.71--57.50) | (259-260) | (2044-2140) | (1142-1198) | (5.9-6.1) | (87-234) | (0-0) | (33.292-52.195) | (0.004-0.008) | (75.11-208.19) |
|  | *%NA* | *100* | *25* | *25* |  |  |  |  |  |  |  |  |  |  |  |
| Sud Lipez | 32 | 6.70 | 8.41 | 93409.00 | -21.67 | -67.85 | 46.03 | 3331.06 | 57.75 | 17.06 | 4312.56 | 0.39 | 5.55 | 0.00 | 35.19 |
|  |  | (1-21) | (6.95-10.38) | (404-421655) | (-22.81--20.14) | (-68.08--67.5) | (36-87) | (3225-3442) | (43-144) | (14.7-17.9) | (3624-4470) | (0-4) | (0.857-94.592) | (0-0.004) | (0.46-473.81) |
|  | *%NA* | *4* | *4* | *4* |  |  |  |  |  |  |  |  |  |  |  |
| Mar Chiquita | 1 | 22.90 | 8.20 | 44865.00 | -30.82 | -62.78 | 183.00 | 4660.00 | 856.00 | 5.90 | 66.00 | 0.00 | 75.77 | 0.01 | 2707.64 |
|  |  | (22.9-22.9) | (8.2-8.2) | (44865-44865) | (-30.82--30.82) | (-62.78--62.78) | (183-183) | (4660-4660) | (856-856) | (5.9-5.9) | (66-66) | (0-0) | (75.77-75.77) | (0.006-0.006) | (2707.64-2707.64) |
|  | *%NA* | - | - | *-* |  |  |  |  |  |  |  |  |  |  |  |

**Appendix S2. Summary of environmental characteristics of lake clusters**

**Figure S2.1** Ranges of the analyzed limnological and geo-climatic data for the lake clusters identified by PCA and cluster analysis. 1: Ecuadorian-Colombian lowlands; 2: Ecuadorian-Colombian Andes; 3: Bolivian-Brazilian lowlands; 4: Perú-Andes; 5: Bolivia-Andes; 6: Southern Altiplano. Lake clusters are arranged by latitude (from lower to higher). The boxes represent the 25th and 75th percentiles, and the median (middle line inside each box).


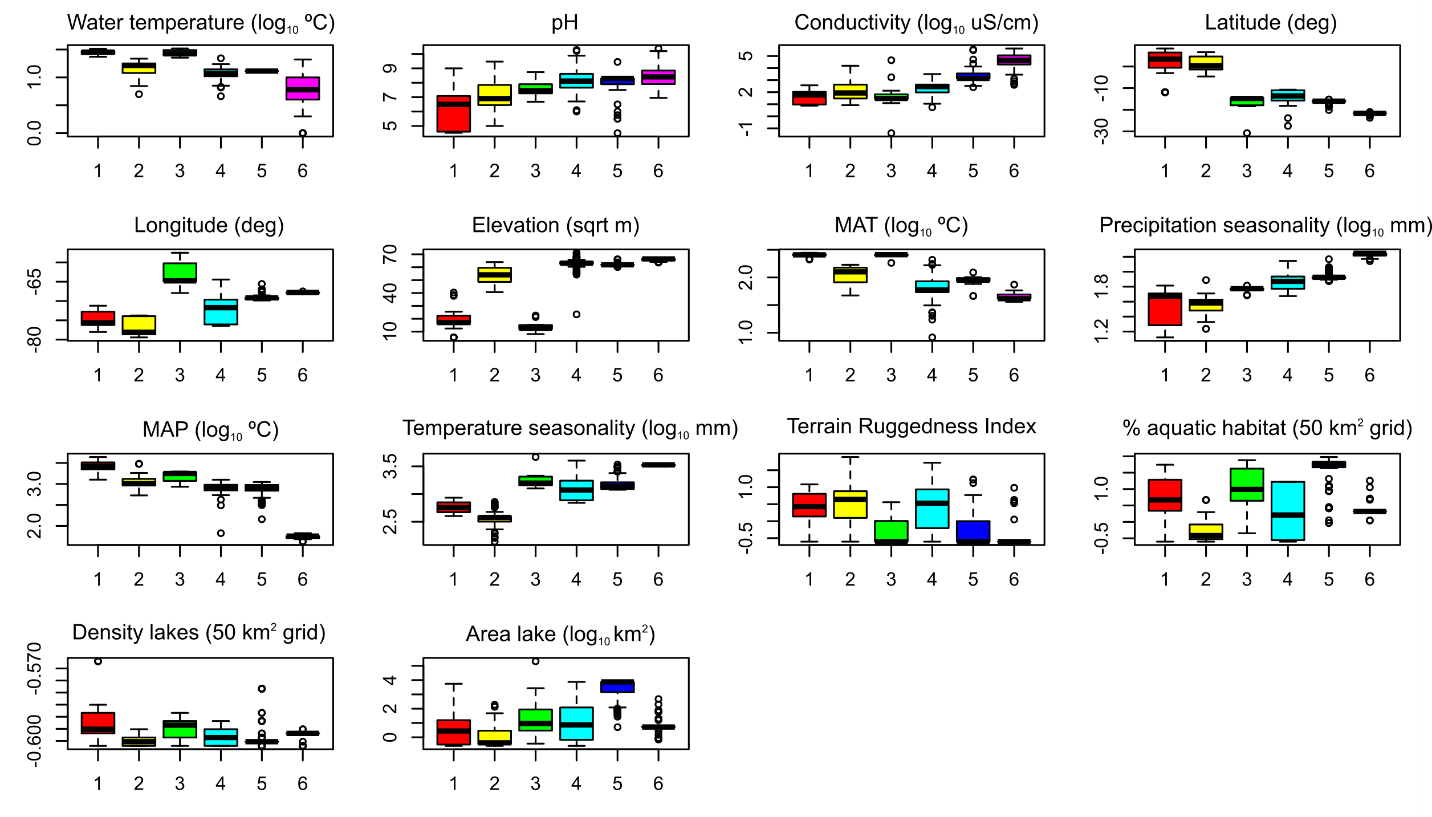


**Figure S2.2** Environmental heterogeneity (left), as expressed as the distance of each site to the centroid (homogeneity of dispersions), and the spatial extent (right) of each lake cluster. Lake clusters are arranged by latitude (from lower to higher). 1: Ecuadorian-Colombian lowlands; 2: Ecuadorian-Colombian Andes; 3: Bolivian-Brazilian lowlands; 4: Perú-Andes; 5: Bolivia-Andes; 6: Southern Altiplano.

**
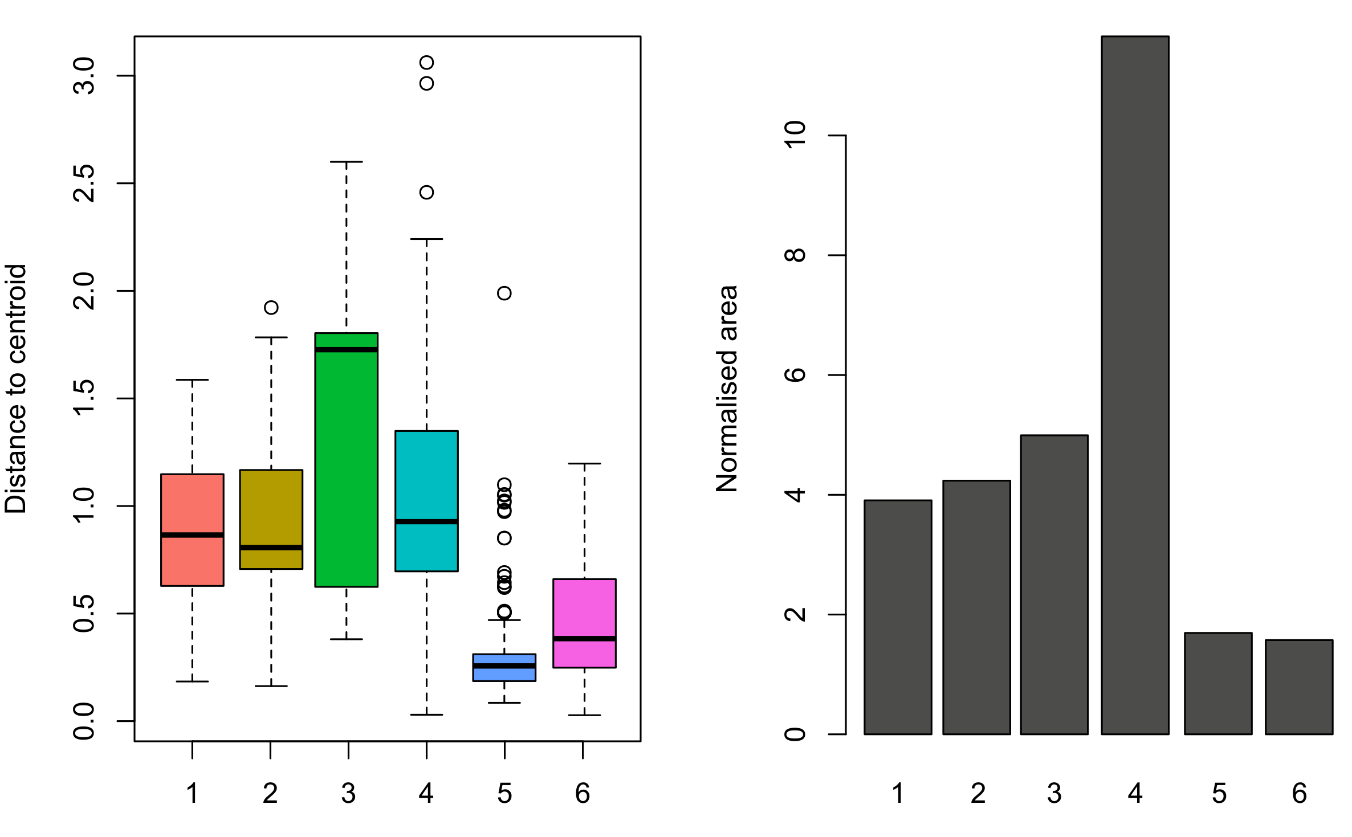
**

**Appendix S3. Assignment of diatom genera to ecological guilds**

**Table S3.1** List of diatom genera identified in lakes of tropical Andes and adjacent lowlands (8ºN–30ºS and 58–79ºW), with the classification of ecological guilds based on Lecointe et al. (1993), Passy (2007), and Rimet and Bouchez (2011). Diatom genera are arranged alphabetically.

| **genera** | **guild** |
| --- | --- |
| *Acanthoceras* | unknown |
| *Achnanthes* | low-profile |
| *Achnanthidium* | low-profile |
| *Actinella* | high-profile |
| *Adlafia* | motile |
| *Amphipleura* | unknown |
| *Amphora* | low-profile |
| *Anomoeoneis* | unknown |
| *Asterionella* | high-profile |
| *Aulacoseira* | high-profile |
| ‘Auxospores’ | unknown |
| *Biremis* | low-profile |
| *Brachysira* | low-profile |
| *Caloneis* | motile |
| *Campylodiscus* | planktic |
| *Cavinula* | motile |
| *Chamaepinnularia* | motile |
| *Cocconeis* | low-profile |
| *Coscinodiscus* | planktic |
| *Cosmioneis* | unknown |
| *Craticula* | motile |
| *Ctenophora* | high-profile |
| *Cyclostephanos* | planktic |
| *Cymatopleura* | motile |
| *Cymbella* | low-profile |
| *Cymbellonitzschia* | motile |
| *Cymbopleura* | low-profile |
| *Denticula* | motile |
| *Desmogonium* | high-profile |
| *Diadesmis* | low-profile |
| *Diatoma* | low-profile |
| *Diploneis* | motile |
| *Discostella* | planktic |
| *Encyonema* | low-profile |
| *Encyonopsis* | low-profile |
| *Entomoneis* | motile |
| *Eolimna* | motile |
| *Epithemia* | motile |
| *Eucocconeis* | low-profile |
| *Eunotia* | high-profile |
| *Eunotioforma* | high-profile |
| *Fallacia* | motile |
| *Fistulifera* | motile |
| *Fragilaria* | high-profile |
| *Fragilariforma* | high-profile |
| *Frankophila* | high-profile |
| *Frustulia* | high-profile |
| *Geissleria* | motile |
| *Gomphonema* | high-profile |
| *Gyrosigma* | motile |
| *Halamphora* | low-profile |
| *Haloroundia* | low-profile |
| *Hannaea* | high-profile |
| *Hantzschia* | motile |
| *Hippodonta* | motile |
| *Humidophila* | unknown |
| *Hungarica* | motile |
| *Karayebia* | low-profile |
| *Kobayasiella* | motile |
| *Lemnicola* | high-profile |
| *Luticola* | motile |
| *Martiana* | low-profile |
| *Mastogloia* | motile |
| *Mayamaea* | motile |
| *Melosira* | planktic |
| *Meridion* | low-profile |
| *Microcostatus* | unknown |
| *Muelleria* | motile |
| *Navicula* | motile |
| *Naviculadicta* | motile |
| *Navicymbula* | low-profile |
| *Neidium* | motile |
| *Nitzschia* | motile |
| *Nupela* | low-profile |
| *Odontidium* | low-profile |
| *Opephora* | high-profile |
| *Orthoseira* | planktic |
| *Paralia* | planktic |
| *Parlibellus* | unknown |
| *Peronia* | unknown |
| *Petroneis* | motile |
| *Pinnularia* | motile |
| *Placoneis* | motile |
| *Plagiotropis* | motile |
| *Planothidium* | low-profile |
| *Platessa* | high-profile |
| *Pleurosigma* | motile |
| *Pleurosira* | planktic |
| *Psammothidium* | low-profile |
| *Pseudostaurosira* | high-profile |
| *Reimeria* | low-profile |
| *Rhoicosphenia* | low-profile |
| *Rhopalodia* | motile |
| *Rossithidium* | low-profile |
| *Scoliopleura* | motile |
| *Sellaphora* | motile |
| *Simonsenia* | motile |
| *Stauroforma* | high-profile |
| *Stauroneis* | motile |
| *Staurophora* | motile |
| *Staurosira* | high-profile |
| *Staurosirella* | high-profile |
| *Stenopterobia* | motile |
| *Stephanodiscus* | planktic |
| *Surirella* | motile |
| *Synedra* | high-profile |
| *Tabellaria* | high-profile |
| *Tabularia* | high-profile |
| *Terpsinoe* | planktic |
| *Tetracyclus* | planktic |
| *Thalassiosira* | planktic |
| *Tryblionella* | motile |
| *Ulnaria* | high-profile |
| Unidentified | unknown |

Proportion of diatom genera classified by ecological guilds

**Appendix S4. Broken stick model results**

**Figure S4.1** Results from Broken stick model: 1) eigenvalues for the first ten PCA axis and the average value; 2) comparison of eigenvalues for PCA axis with the broken stick model. Broken stick values above eigenvalues mean non-random variability.

**
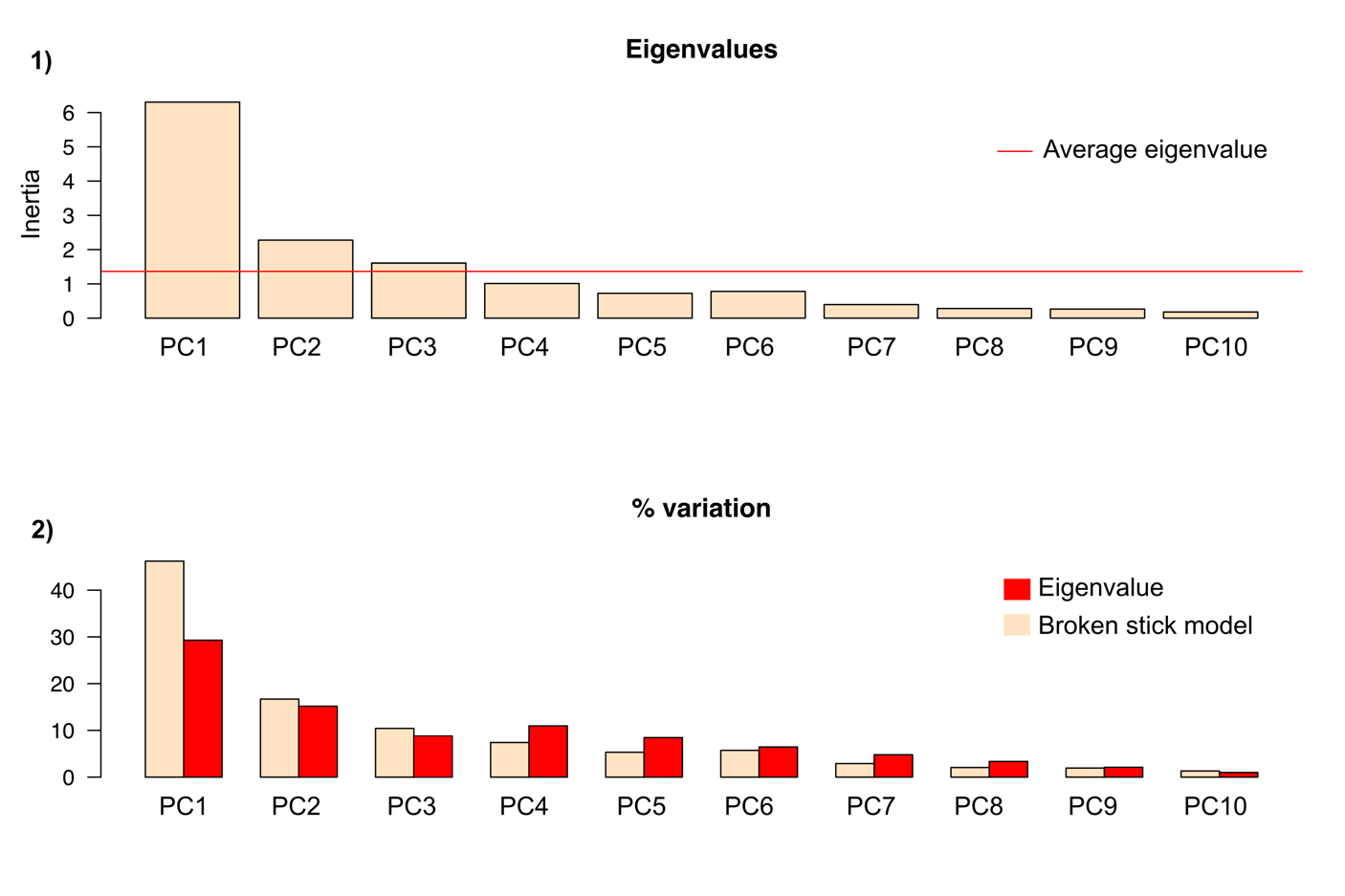
**

**Appendix S5. Supporting non-metric multidimensional scaling results**

**Fig. S5.1** Results of Non-Metric Multidimensional Scaling (NMDS) with Chao distance measure on Hellinger transformed presence-absence diatom matrix for the surface sediment lake samples (2D stress=0.17). Left: sample scores labeled by regions with environmental variable fitting showing the statistically significant variables; the length of each vector is proportional to the correlation between variables and NMDS axes. Right: species scores with the 20% most abundant species showing 50% best environmental fit in the first two NMDS axes.


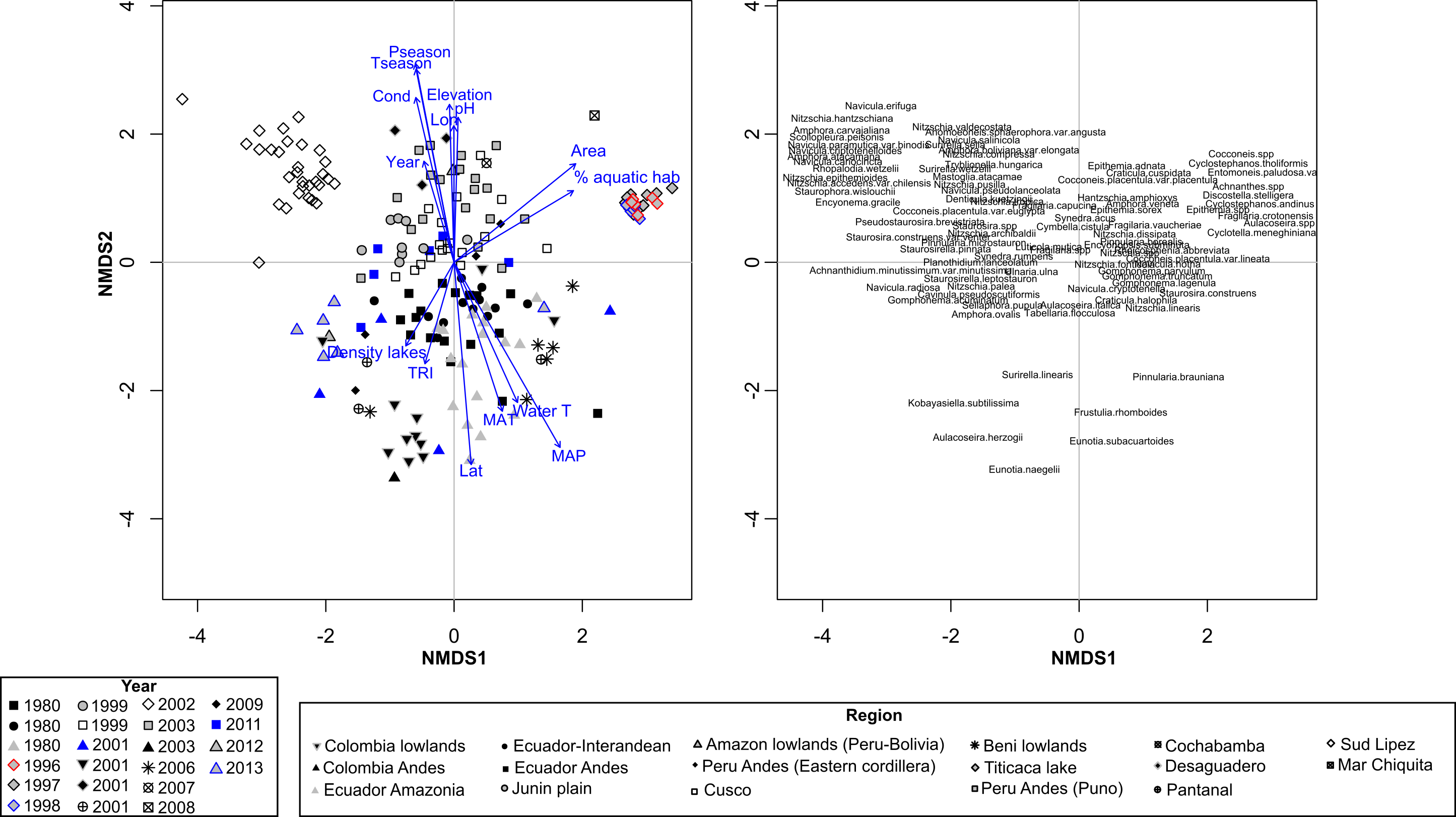


**Fig. S5.2** Results of Non-Metric Multidimensional Scaling (NMDS) with Chao distance measure on Hellinger transformed presence-absence diatom matrix for the periphyton lake samples (2D stress=0.18). Left: sample scores labeled by regions with environmental variable fitting showing the statistically significant variables; the length of each vector is proportional to the correlation between variables and NMDS axes. Right: species scores with the 20% most abundant species showing 50% best environmental fit in the first two NMDS axes.


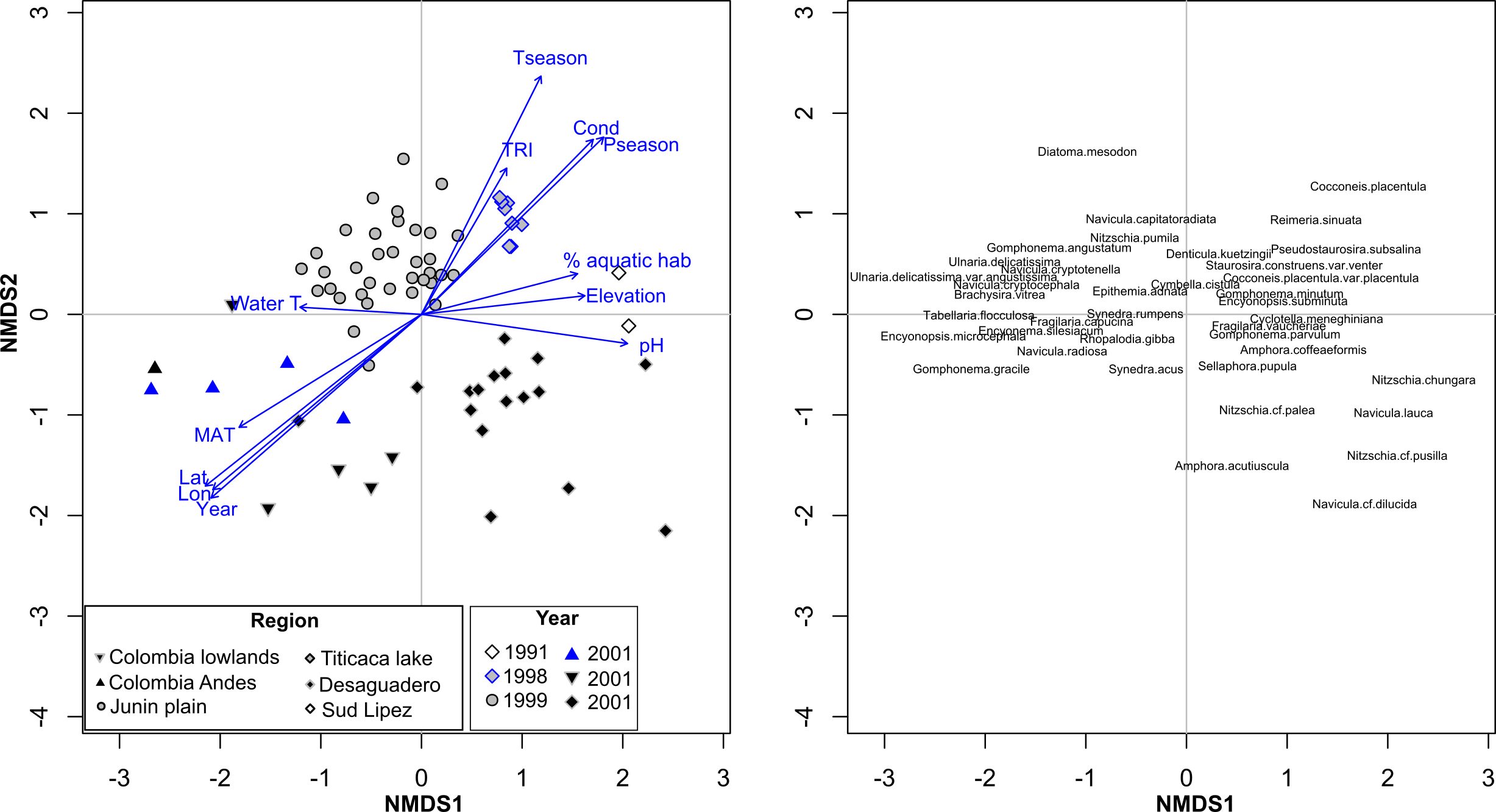


**Fig. S5.3** Results of Non-Metric Multidimensional Scaling (NMDS) with Chao distance measure on Hellinger transformed presence-absence diatom matrix for the plankton lake samples (2D stress=0.19). Left: Left: sample scores labeled by regions with environmental variable fitting showing the statistically significant variables; the length of each vector is proportional to the correlation between variables and NMDS axes. Right: species scores with the 20% most abundant species showing 50% best environmental fit in the first two NMDS axes.


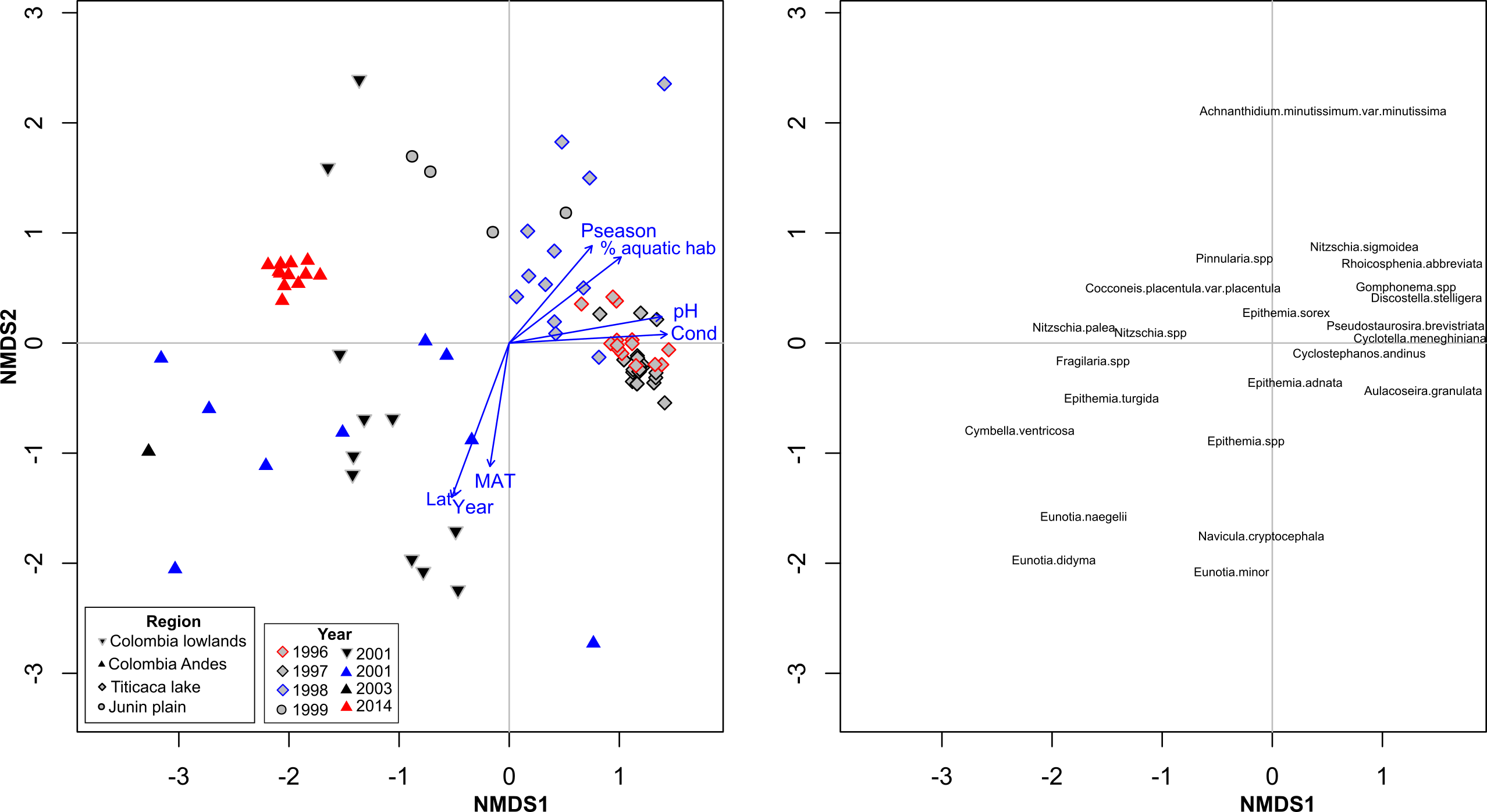


**Appendix S6. Supporting statistical results of Redundancy Analysis and variance partitioning**

**Table S5.1** Results of RDA with forward selection on the three set of predictors (environment, geographic and topographic) using the two stop criteria to account for different number of predictors in each forward selection procedure (i.e. one for each set of predictors). R^2^ full model was run including all variables. Matrix: subset of diatom species data (all and for each ecological guild separately: high-profile, low-profile, motile and planktonic). ENV_PC = forward-selected variables of the environmental component (three first axes of the Principal Components Analysis); G_PCNM = forward-selected variables of the geographic component (pairwise Euclidean distances); T_PCNM = forward-selected variables of the topographic component (pairwise resistance distances).

| **Cluster** | **Name** | **Matrix** | **Predictors** | **F** | ***p*** | **R^2^ full model** | **Adj. R^2^** | **Forward-selected variables** |
| --- | --- | --- | --- | --- | --- | --- | --- | --- |
| 1 | Ecuadorean-Colombian lowlands | all species | Environment | 1.716 | 0.001 | 0.080 |  |  |
| 1 | Ecuadorean-Colombian lowlands | all species |  | 2.059 | 0.001 |  | 0.036 | ENV_PC1 |
| 1 | Ecuadorean-Colombian lowlands | all species |  | 1.545 | 0.004 |  | 0.055 | ENV_PC2 |
| 1 | Ecuadorean-Colombian lowlands | all species |  | 1.682 | 0.003 |  | 0.080 | ENV_PC3 |
| 1 | Ecuadorean-Colombian lowlands | all species | Geographic | 1.555 | 0.001 | 0.106 |  |  |
| 1 | Ecuadorean-Colombian lowlands | all species |  |  |  |  | 0.034 | G_PCNM3 |
| 1 | Ecuadorean-Colombian lowlands | all species |  |  |  |  | 0.070 | G_PCNM1 |
| 1 | Ecuadorean-Colombian lowlands | all species |  |  |  |  | 0.094 | G_PCNM4 |
| 1 | Ecuadorean-Colombian lowlands | all species | Topographic | 1.184 | 0.001 | 0.044 |  |  |
| 1 | Ecuadorean-Colombian lowlands | all species |  | 1.727 | 0.002 |  | 0.025 | T_PCNM2 |
| 1 | Ecuadorean-Colombian lowlands | high-profile | Environment | 4.027 | 0.001 | 0.245 |  |  |
| 1 | Ecuadorean-Colombian lowlands | high-profile |  | 7.819 | 0.001 |  | 0.196 | ENV_PC3 |
| 1 | Ecuadorean-Colombian lowlands | high-profile |  | 2.517 | 0.013 |  | 0.239 | ENV_PC1 |
| 1 | Ecuadorean-Colombian lowlands | high-profile | Geographic | 4.462 | 0.001 | 0.426 |  |  |
| 1 | Ecuadorean-Colombian lowlands | high-profile |  | 6.420 | 0.002 |  | 0.162 | G_PCNM1 |
| 1 | Ecuadorean-Colombian lowlands | high-profile |  | 6.847 | 0.002 |  | 0.311 | G_PCNM3 |
| 1 | Ecuadorean-Colombian lowlands | high-profile |  | 3.163 | 0.008 |  | 0.364 | G_PCNM6 |
| 1 | Ecuadorean-Colombian lowlands | high-profile |  | 2.651 | 0.016 |  | 0.404 | G_PCNM4 |
| 1 | Ecuadorean-Colombian lowlands | high-profile | Topographic | 1.621 | 0.023 | 0.134 |  |  |
| 1 | Ecuadorean-Colombian lowlands | high-profile |  | 5.651 | 0.001 |  | 0.142 | T_PCNM2 |
| 1 | Ecuadorean-Colombian lowlands | low-profile | Environment | 5.598 | 0.001 | 0.330 |  |  |
| 1 | Ecuadorean-Colombian lowlands | low-profile |  | 10.078 | 0.001 |  | 0.245 | ENV_PC3 |
| 1 | Ecuadorean-Colombian lowlands | low-profile |  | 2.574 | 0.008 |  | 0.286 | ENV_PC1 |
| 1 | Ecuadorean-Colombian lowlands | low-profile |  | 2.692 | 0.007 |  | 0.330 | ENV_PC2 |
| 1 | Ecuadorean-Colombian lowlands | low-profile | Geographic | 5.715 | 0.001 | 0.503 |  |  |
| 1 | Ecuadorean-Colombian lowlands | low-profile |  | 11.225 | 0.001 |  | 0.267 | G_PCNM1 |
| 1 | Ecuadorean-Colombian lowlands | low-profile |  | 8.487 | 0.002 |  | 0.427 | G_PCNM3 |
| 1 | Ecuadorean-Colombian lowlands | low-profile | Topographic | 2.250 | 0.004 | 0.238 |  |  |
| 1 | Ecuadorean-Colombian lowlands | low-profile |  | 5.275 | 0.002 |  | 0.132 | T_PCNM2 |
| 1 | Ecuadorean-Colombian lowlands | low-profile |  | 3.588 | 0.009 |  | 0.208 | T_PCNM1 |
| 1 | Ecuadorean-Colombian lowlands | motile | Environment | 2.898 | 0.001 | 0.169 |  |  |
| 1 | Ecuadorean-Colombian lowlands | motile |  | 5.844 | 0.002 |  | 0.147 | ENV_PC1 |
| 1 | Ecuadorean-Colombian lowlands | motile | Geographic | 3.586 | 0.001 | 0.357 |  |  |
| 1 | Ecuadorean-Colombian lowlands | motile |  |  |  |  | 0.135 | G_PCNM3 |
| 1 | Ecuadorean-Colombian lowlands | motile |  |  |  |  | 0.237 | G_PCNM1 |
| 1 | Ecuadorean-Colombian lowlands | motile |  |  |  |  | 0.282 | G_PCNM4 |
| 1 | Ecuadorean-Colombian lowlands | motile | Topographic | 2.111 | 0.001 | 0.217 |  |  |
| 1 | Ecuadorean-Colombian lowlands | motile |  | 3.303 | 0.009 |  | 0.076 | T_PCNM2 |
| 1 | Ecuadorean-Colombian lowlands | motile |  | 3.050 | 0.006 |  | 0.141 | T_PCNM1 |
| 1 | Ecuadorean-Colombian lowlands | motile |  | 2.483 | 0.023 |  | 0.188 | T_PCNM5 |
| 1 | Ecuadorean-Colombian lowlands | planktonic | Environment | 5.033 | 0.001 | 0.302 |  |  |
| 1 | Ecuadorean-Colombian lowlands | planktonic |  | 9.645 | 0.001 |  | 0.236 | ENV_PC3 |
| 1 | Ecuadorean-Colombian lowlands | planktonic |  | 2.459 | 0.023 |  | 0.275 | ENV_PC1 |
| 1 | Ecuadorean-Colombian lowlands | planktonic | Geographic | 8.224 | 0.001 | 0.608 |  |  |
| 1 | Ecuadorean-Colombian lowlands | planktonic |  | 7.361 | 0.001 |  | 0.185 | G_PCNM3 |
| 1 | Ecuadorean-Colombian lowlands | planktonic |  | 8.973 | 0.001 |  | 0.371 | G_PCNM1 |
| 1 | Ecuadorean-Colombian lowlands | planktonic |  | 4.501 | 0.006 |  | 0.446 | G_PCNM5 |
| 1 | Ecuadorean-Colombian lowlands | planktonic |  | 5.240 | 0.002 |  | 0.526 | G_PCNM6 |
| 1 | Ecuadorean-Colombian lowlands | planktonic |  | 5.807 | 0.001 |  | 0.605 | G_PCNM4 |
| 1 | Ecuadorean-Colombian lowlands | planktonic | Topographic | 2.106 | 0.005 | 0.217 |  |  |
| 1 | Ecuadorean-Colombian lowlands | planktonic |  | 4.676 | 0.004 |  | 0.116 | T_PCNM2 |
| 2 | Ecuadorean-Colombian Andes | all species | Environment | 1.384 | 0.001 | 0.027 |  |  |
| 2 | Ecuadorean-Colombian Andes | all species |  | 1.670 | 0.002 | 0.016 |  | ENV_PC2 |
| 2 | Ecuadorean-Colombian Andes | all species |  | 1.283 | 0.033 | 0.022 |  | ENV_PC3 |
| 2 | Ecuadorean-Colombian Andes | all species | Geographic | 1.526 | 0.001 | 0.071 |  |  |
| 2 | Ecuadorean-Colombian Andes | all species |  | 2.679 | 0.001 |  | 0.038 | G_PCNM1 |
| 2 | Ecuadorean-Colombian Andes | all species |  | 1.757 | 0.001 |  | 0.056 | G_PCNM3 |
| 2 | Ecuadorean-Colombian Andes | all species |  | 1.394 | 0.002 |  | 0.065 | G_PCNM5 |
| 2 | Ecuadorean-Colombian Andes | all species | Topographic | 1.193 | 0.009 | 0.014 |  |  |
| 2 | Ecuadorean-Colombian Andes | all species |  | 1.636 | 0.002 |  | 0.014 | T_PCNM2 |
| 2 | Ecuadorean-Colombian Andes | high-profile | Environment | 3.304 | 0.001 | 0.141 |  |  |
| 2 | Ecuadorean-Colombian Andes | high-profile |  | 5.514 | 0.001 |  | 0.097 | ENV_PC2 |
| 2 | Ecuadorean-Colombian Andes | high-profile |  | 2.222 | 0.013 |  | 0.123 | ENV_PC3 |
| 2 | Ecuadorean-Colombian Andes | high-profile |  | 1.845 | 0.046 |  | 0.141 | ENV_PC1 |
| 2 | Ecuadorean-Colombian Andes | high-profile | Geographic | 3.191 | 0.001 | 0.239 |  |  |
| 2 | Ecuadorean-Colombian Andes | high-profile |  | 9.839 | 0.001 |  | 0.174 | G_PCNM1 |
| 2 | Ecuadorean-Colombian Andes | high-profile |  | 2.414 | 0.008 |  | 0.201 | G_PCNM3 |
| 2 | Ecuadorean-Colombian Andes | high-profile |  | 1.943 | 0.024 |  | 0.220 | G_PCNM4 |
| 2 | Ecuadorean-Colombian Andes | high-profile |  | 1.982 | 0.031 |  | 0.239 | G_PCNM6 |
| 2 | Ecuadorean-Colombian Andes | high-profile | Topographic | 2.518 | 0.001 | 0.098 |  |  |
| 2 | Ecuadorean-Colombian Andes | high-profile |  | 5.220 | 0.002 |  | 0.091 | T_PCNM2 |
| 2 | Ecuadorean-Colombian Andes | low-profile | Environment | 1.727 | 0.041 | 0.049 |  |  |
| 2 | Ecuadorean-Colombian Andes | low-profile |  | 2.641 | 0.028 |  | 0.038 | ENV_PC3 |
| 2 | Ecuadorean-Colombian Andes | low-profile | Geographic | 1.888 | 0.005 | 0.113 |  |  |
| 2 | Ecuadorean-Colombian Andes | low-profile |  | 5.623 | 0.002 |  | 0.099 | G_PCNM1 |
| 2 | Ecuadorean-Colombian Andes | low-profile | Topographic | 1.458 | 0.073 | 0.032 |  |  |
| 2 | Ecuadorean-Colombian Andes | motile | Environment | 2.159 | 0.011 | 0.076 |  |  |
| 2 | Ecuadorean-Colombian Andes | motile |  | 2.509 | 0.026 |  | 0.035 | ENV_PC1 |
| 2 | Ecuadorean-Colombian Andes | motile |  | 2.671 | 0.017 |  | 0.072 | ENV_PC3 |
| 2 | Ecuadorean-Colombian Andes | motile | Geographic | 2.508 | 0.001 | 0.178 |  |  |
| 2 | Ecuadorean-Colombian Andes | motile |  | 5.501 | 0.002 |  | 0.097 | G_PCNM1 |
| 2 | Ecuadorean-Colombian Andes | motile |  | 3.293 | 0.010 |  | 0.145 | G_PCNM5 |
| 2 | Ecuadorean-Colombian Andes | motile | Topographic | 1.281 | 0.126 | 0.020 |  |  |
| 2 | Ecuadorean-Colombian Andes | planktonic | Environment | 3.215 | 0.001 | 0.137 |  |  |
| 2 | Ecuadorean-Colombian Andes | planktonic |  | 4.354 | 0.001 |  | 0.074 | ENV_PC2 |
| 2 | Ecuadorean-Colombian Andes | planktonic |  | 3.009 | 0.003 |  | 0.117 | ENV_PC3 |
| 2 | Ecuadorean-Colombian Andes | planktonic |  | 1.898 | 0.030 |  | 0.137 | ENV_PC1 |
| 2 | Ecuadorean-Colombian Andes | planktonic | Geographic | 2.958 | 0.001 | 0.219 |  |  |
| 2 | Ecuadorean-Colombian Andes | planktonic |  | 10.580 | 0.002 |  | 0.186 | G_PCNM1 |
| 2 | Ecuadorean-Colombian Andes | planktonic |  | 2.506 | 0.012 |  | 0.215 | G_PCNM4 |
| 2 | Ecuadorean-Colombian Andes | planktonic | Topographic | 2.276 | 0.006 | 0.084 |  |  |
| 2 | Ecuadorean-Colombian Andes | planktonic |  | 4.421 | 0.002 |  | 0.075 | T_PCNM2 |
| 3 | Bolivian-Brazilian lowlands | all species | Environment | 1.122 | 0.142 | 0.032 |  |  |
| 3 | Bolivian-Brazilian lowlands | all species | Geographic | 1.182 | 0.049 | 0.047 |  |  |
| 3 | Bolivian-Brazilian lowlands | all species |  | 1.281 | 0.025 |  | 0.093 | G_PCNM1 |
| 3 | Bolivian-Brazilian lowlands | all species | Topographic | 1.259 | 0.026 | 0.070 |  |  |
| 3 | Bolivian-Brazilian lowlands | all species |  | 1.331 | 0.036 |  | 0.029 | T_PCNM3 |
| 3 | Bolivian-Brazilian lowlands | high-profile | Environment | 1.415 | 0.046 | 0.102 |  |  |
| 3 | Bolivian-Brazilian lowlands | high-profile |  | 2.016 | 0.005 |  | 0.085 | ENV_PC2 |
| 3 | Bolivian-Brazilian lowlands | high-profile |  | 1.678 | 0.038 |  | 0.143 | ENV_PC1 |
| 3 | Bolivian-Brazilian lowlands | high-profile | Geographic | 1.539 | 0.020 | 0.128 |  |  |
| 3 | Bolivian-Brazilian lowlands | high-profile |  | 1.748 | 0.029 |  | 0.064 | G_PCNM3 |
| 3 | Bolivian-Brazilian lowlands | high-profile | Topographic | 1.892 | 0.005 | 0.196 |  |  |
| 3 | Bolivian-Brazilian lowlands | high-profile |  | 2.911 | 0.008 |  | 0.148 | T_PCNM1 |
| 3 | Bolivian-Brazilian lowlands | low-profile | Environment | 1.431 | 0.114 | 0.073 |  |  |
| 3 | Bolivian-Brazilian lowlands | low-profile | Geographic | 1.516 | 0.042 | 0.123 |  |  |
| 3 | Bolivian-Brazilian lowlands | low-profile |  | 1.687 | 0.082 |  | 0.059 | G_PCNM3 |
| 3 | Bolivian-Brazilian lowlands | low-profile | Topographic | 1.848 | 0.009 | 0.188 |  |  |
| 3 | Bolivian-Brazilian lowlands | low-profile |  | 2.380 | 0.010 |  | 0.111 | T_PCNM1 |
| 3 | Bolivian-Brazilian lowlands | motile | Environment | 1.357 | 0.032 | 0.069 |  |  |
| 3 | Bolivian-Brazilian lowlands | motile |  | 1.503 | 0.046 |  | 0.044 | PC2 |
| 3 | Bolivian-Brazilian lowlands | motile | Geographic | 1.224 | 0.100 | 0.058 |  |  |
| 3 | Bolivian-Brazilian lowlands | motile | Topographic | 1.564 | 0.003 | 0.134 |  |  |
| 3 | Bolivian-Brazilian lowlands | motile |  | 2.127 | 0.010 |  | 0.093 | T_PCNM1 |
| 3 | Bolivian-Brazilian lowlands | planktonic | Environment | 1.017 | 0.438 | 0.003 |  |  |
| 3 | Bolivian-Brazilian lowlands | planktonic | Geographic | 1.348 | 0.185 | 0.086 |  |  |
| 3 | Bolivian-Brazilian lowlands | planktonic | Topographic | 8.695 | 0.006 | 0.875 |  |  |
| 3 | Bolivian-Brazilian lowlands | planktonic |  | 1.893 | 0.062 |  | 0.075 | T_PCNM4 |
| 3 | Bolivian-Brazilian lowlands | planktonic |  | 2.071 | 0.048 |  | 0.165 | T_PCNM6 |
| 4 | Peruvian Andes | all species | Environment | 2.984 | 0.001 | 0.066 |  |  |
| 4 | Peruvian Andes | all species |  | 3.736 | 0.001 |  | 0.032 | ENV_PC3 |
| 4 | Peruvian Andes | all species |  | 2.479 | 0.001 |  | 0.050 | ENV_PC2 |
| 4 | Peruvian Andes | all species |  | 2.429 | 0.001 |  | 0.066 | ENV_PC1 |
| 4 | Peruvian Andes | all species | Geographic | 1.548 | 0.001 | 0.091 |  |  |
| 4 | Peruvian Andes | all species |  | 4.495 | 0.001 |  | 0.041 | G_PCNM1 |
| 4 | Peruvian Andes | all species |  | 2.553 | 0.001 |  | 0.059 | G_PCNM2 |
| 4 | Peruvian Andes | all species |  | 1.646 | 0.001 |  | 0.066 | G_PCNM4 |
| 4 | Peruvian Andes | all species |  | 1.614 | 0.001 |  | 0.074 | G_PCNM11 |
| 4 | Peruvian Andes | all species |  | 1.484 | 0.003 |  | 0.079 | G_PCNM5 |
| 4 | Peruvian Andes | all species |  | 1.446 | 0.005 |  | 0.085 | G_PCNM10 |
| 4 | Peruvian Andes | all species |  | 1.438 | 0.004 |  | 0.090 | G_PCNM3 |
| 4 | Peruvian Andes | all species | Topographic | 1.341 | 0.001 | 0.088 |  |  |
| 4 | Peruvian Andes | all species |  | 3.173 | 0.001 |  | 0.026 | T_PCNM1 |
| 4 | Peruvian Andes | all species |  | 1.964 | 0.001 |  | 0.037 | T_PCNM7 |
| 4 | Peruvian Andes | all species |  | 1.844 | 0.001 |  | 0.047 | T_PCNM2 |
| 4 | Peruvian Andes | all species |  | 1.517 | 0.001 |  | 0.054 | T_PCNM4 |
| 4 | Peruvian Andes | all species |  | 1.517 | 0.003 |  | 0.060 | T_PCNM22 |
| 4 | Peruvian Andes | all species |  | 1.386 | 0.006 |  | 0.064 | T_PCNM3 |
| 4 | Peruvian Andes | all species |  | 1.330 | 0.012 |  | 0.068 | T_PCNM8 |
| 4 | Peruvian Andes | all species |  | 1.324 | 0.020 |  | 0.072 | T_PCNM9 |
| 4 | Peruvian Andes | all species |  | 1.297 | 0.021 |  | 0.076 | T_PCNM6 |
| 4 | Peruvian Andes | all species |  | 1.271 | 0.035 |  | 0.080 | T_PCNM11 |
| 4 | Peruvian Andes | high-profile | Environment | 2.300 | 0.001 | 0.031 |  |  |
| 4 | Peruvian Andes | high-profile |  | 3.168 | 0.001 |  | 0.026 | ENV_PC3 |
| 4 | Peruvian Andes | high-profile |  | 1.996 | 0.010 |  | 0.038 | ENV_PC2 |
| 4 | Peruvian Andes | high-profile | Geographic | 1.394 | 0.001 | 0.067 |  |  |
| 4 | Peruvian Andes | high-profile |  | 3.231 | 0.004 | 0.026 |  | G_PCNM1 |
| 4 | Peruvian Andes | high-profile |  | 3.152 | 0.002 | 0.052 |  | G_PCNM2 |
| 4 | Peruvian Andes | high-profile |  | 2.020 | 0.006 | 0.064 |  | G_PCNM13 |
| 4 | Peruvian Andes | high-profile | Topographic | 1.267 | 0.002 | 0.081 |  |  |
| 4 | Peruvian Andes | high-profile |  | 2.992 | 0.001 |  | 0.024 | T_PCNM1 |
| 4 | Peruvian Andes | high-profile |  | 2.799 | 0.001 |  | 0.045 | T_PCNM22 |
| 4 | Peruvian Andes | high-profile |  | 2.349 | 0.002 |  | 0.061 | T_PCNM2 |
| 4 | Peruvian Andes | high-profile |  | 1.578 | 0.035 |  | 0.068 | T_PCNM11 |
| 4 | Peruvian Andes | low-profile | Environment | 1.640 | 0.005 | 0.015 |  |  |
| 4 | Peruvian Andes | low-profile |  | 1.884 | 0.016 |  | 0.011 | PC1 |
| 4 | Peruvian Andes | low-profile | Geographic | 1.209 | 0.010 | 0.037 |  |  |
| 4 | Peruvian Andes | low-profile |  | 3.045 | 0.002 |  | 0.024 | G_PCNM1 |
| 4 | Peruvian Andes | low-profile |  | 1.569 | 0.044 |  | 0.031 | G_PCNM2 |
| 4 | Peruvian Andes | low-profile | Topographic | 1.084 | 0.125 | 0.027 |  |  |
| 4 | Peruvian Andes | motile | Environment | 2.421 | 0.001 | 0.033 |  |  |
| 4 | Peruvian Andes | motile |  | 2.719 | 0.002 |  | 0.021 | ENV_PC2 |
| 4 | Peruvian Andes | motile |  | 1.900 | 0.003 |  | 0.031 | ENV_PC3 |
| 4 | Peruvian Andes | motile | Geographic | 1.296 | 0.001 | 0.051 |  |  |
| 4 | Peruvian Andes | motile |  | 2.801 | 0.001 |  | 0.021 | G_PCNM1 |
| 4 | Peruvian Andes | motile |  | 2.427 | 0.001 |  | 0.038 | G_PCNM2 |
| 4 | Peruvian Andes | motile |  | 2.004 | 0.004 |  | 0.050 | G_PCNM3 |
| 4 | Peruvian Andes | motile | Topographic | 1.246 | 0.001 | 0.075 |  |  |
| 4 | Peruvian Andes | motile |  | 2.454 | 0.003 |  | 0.017 | T_PCNM1 |
| 4 | Peruvian Andes | motile |  | 2.079 | 0.003 |  | 0.030 | T_PCNM22 |
| 4 | Peruvian Andes | motile |  | 1.869 | 0.003 |  | 0.041 | T_PCNM2 |
| 4 | Peruvian Andes | motile |  | 1.751 | 0.013 |  | 0.050 | T_PCNM8 |
| 4 | Peruvian Andes | motile |  | 1.721 | 0.006 |  | 0.059 | T_PCNM26 |
| 4 | Peruvian Andes | motile |  | 1.541 | 0.022 |  | 0.065 | T_PCNM7 |
| 4 | Peruvian Andes | motile |  | 1.506 | 0.028 |  | 0.071 | T_PCNM11 |
| 4 | Peruvian Andes | planktonic | Environment | 2.895 | 0.001 | 0.044 |  |  |
| 4 | Peruvian Andes | planktonic |  | 5.184 | 0.001 |  | 0.044 | PC2 |
| 4 | Peruvian Andes | planktonic | Geographic | 1.240 | 0.079 | 0.042 |  |  |
| 4 | Peruvian Andes | planktonic | Topographic | 1.622 | 0.001 | 0.170 |  |  |
| 4 | Peruvian Andes | planktonic |  | 3.169 | 0.006 |  | 0.026 | T_PCNM22 |
| 4 | Peruvian Andes | planktonic |  | 2.995 | 0.010 |  | 0.049 | T_PCNM2 |
| 4 | Peruvian Andes | planktonic |  | 2.704 | 0.008 |  | 0.069 | T_PCNM1 |
| 4 | Peruvian Andes | planktonic |  | 2.731 | 0.011 |  | 0.089 | T_PCNM8 |
| 4 | Peruvian Andes | planktonic |  | 2.663 | 0.009 |  | 0.108 | T_PCNM7 |
| 4 | Peruvian Andes | planktonic |  | 2.654 | 0.011 |  | 0.127 | T_PCNM11 |
| 5 | Bolivian Andes | all species | Environment | 5.615 | 0.001 | 0.206 |  |  |
| 5 | Bolivian Andes | all species |  | 16.182 | 0.001 |  | 0.174 | ENV_PC3 |
| 5 | Bolivian Andes | all species |  | 2.866 | 0.005 |  | 0.195 | ENV_PC2 |
| 5 | Bolivian Andes | all species |  | 1.903 | 0.024 |  | 0.206 | ENV_PC1 |
| 5 | Bolivian Andes | all species | Geographic | 3.239 | 0.001 | 0.303 |  |  |
| 5 | Bolivian Andes | all species | Geographic | 13.509 | 0.001 |  | 0.148 | G_PCNM1 |
| 5 | Bolivian Andes | all species | Geographic | 2.354 | 0.014 |  | 0.164 | G_PCNM9 |
| 5 | Bolivian Andes | all species | Geographic | 2.327 | 0.015 |  | 0.180 | G_PCNM2 |
| 5 | Bolivian Andes | all species | Geographic | 2.327 | 0.009 |  | 0.195 | G_PCNM5 |
| 5 | Bolivian Andes | all species | Geographic | 2.091 | 0.014 |  | 0.208 | G_PCNM6 |
| 5 | Bolivian Andes | all species | Geographic | 1.948 | 0.030 |  | 0.219 | G_PCNM10 |
| 5 | Bolivian Andes | all species | Geographic | 1.929 | 0.028 |  | 0.230 | G_PCNM12 |
| 5 | Bolivian Andes | all species | Geographic | 1.918 | 0.037 |  | 0.240 | G_PCNM4 |
| 5 | Bolivian Andes | all species | Topographic | 4.710 | 0.001 | 0.134 |  |  |
| 5 | Bolivian Andes | all species |  | 9.170 | 0.001 |  | 0.112 | T_PCNM2 |
| 5 | Bolivian Andes | high-profile | Environment | 2.461 | 0.001 | 0.039 |  |  |
| 5 | Bolivian Andes | high-profile |  | 3.201 | 0.002 |  | 0.030 | PC1 |
| 5 | Bolivian Andes | high-profile |  | 1.691 | 0.014 |  | 0.039 | PC2 |
| 5 | Bolivian Andes | high-profile | Geographic | 1.461 | 0.001 | 0.082 |  |  |
| 5 | Bolivian Andes | high-profile |  | 6.604 | 0.002 |  | 0.072 | G_PCNM1 |
| 5 | Bolivian Andes | high-profile | Topographic | 2.481 | 0.001 | 0.058 |  |  |
| 5 | Bolivian Andes | high-profile |  | 4.566 | 0.002 |  | 0.047 | T_PCNM2 |
| 5 | Bolivian Andes | low-profile | Environment | 2.471 | 0.001 | 0.058 |  |  |
| 5 | Bolivian Andes | low-profile |  | 4.447 | 0.002 |  | 0.046 | ENV_PC3 |
| 5 | Bolivian Andes | low-profile |  | 1.722 | 0.016 |  | 0.055 | ENV_PC2 |
| 5 | Bolivian Andes | low-profile | Geographic | 1.553 | 0.001 | 0.097 |  |  |
| 5 | Bolivian Andes | low-profile |  | 5.901 | 0.001 |  | 0.064 | G_PCNM1 |
| 5 | Bolivian Andes | low-profile |  | 2.160 | 0.002 |  | 0.079 | G_PCNM2 |
| 5 | Bolivian Andes | low-profile |  | 1.882 | 0.007 |  | 0.090 | G_PCNM3 |
| 5 | Bolivian Andes | low-profile | Topographic | 2.770 | 0.001 | 0.069 |  |  |
| 5 | Bolivian Andes | low-profile |  | 4.169 | 0.001 |  | 0.042 | T_PCNM2 |
| 5 | Bolivian Andes | low-profile |  | 2.932 | 0.001 |  | 0.068 | T_PCNM3 |
| 5 | Bolivian Andes | motile | Environment | 1.901 | 0.001 | 0.024 |  |  |
| 5 | Bolivian Andes | motile |  | 2.238 | 0.002 |  | 0.017 | PC1 |
| 5 | Bolivian Andes | motile |  | 1.547 | 0.012 |  | 0.024 | PC2 |
| 5 | Bolivian Andes | motile | Geographic | 1.404 | 0.001 | 0.073 |  |  |
| 5 | Bolivian Andes | motile |  | 4.141 | 0.001 |  | 0.042 | G_PCNM1 |
| 5 | Bolivian Andes | motile |  | 3.223 | 0.001 |  | 0.071 | G_PCNM2 |
| 5 | Bolivian Andes | motile | Topographic | 2.378 | 0.001 | 0.054 |  |  |
| 5 | Bolivian Andes | motile |  | 3.147 | 0.001 |  | 0.029 | T_PCNM2 |
| 5 | Bolivian Andes | motile |  | 2.904 | 0.001 |  | 0.054 | T_PCNM3 |
| 5 | Bolivian Andes | planktonic | Environment | 1.515 | 0.068 | 0.021 |  |  |
| 5 | Bolivian Andes | planktonic |  | 3.151 | 0.001 |  | 0.015 | ENV_PC3 |
| 5 | Bolivian Andes | planktonic | Geographic | 1.119 | 0.300 | 0.023 |  |  |
| 5 | Bolivian Andes | planktonic | Topographic | 0.673 | 0.770 | 0.010 |  |  |
| 6 | Southern Altiplano | all species | Environment | 1.387 | 0.025 | 0.056 |  |  |
| 6 | Southern Altiplano | all species |  | 1.567 | 0.024 |  | 0.042 | PC1 |
| 6 | Southern Altiplano | all species | Geographic | 1.412 | 0.001 | 0.160 |  |  |
| 6 | Southern Altiplano | all species |  | 1.481 | 0.030 |  | 0.036 | G_PCNM3 |
| 6 | Southern Altiplano | all species |  | 1.519 | 0.048 |  | 0.076 | G_PCNM5 |
| 6 | Southern Altiplano | all species | Topographic | 1.016 | 0.420 | 0.005 |  |  |
| 6 | Southern Altiplano | high-profile | Environment | 0.717 | 0.919 | 0.001 |  |  |
| 6 | Southern Altiplano | high-profile | Geographic | 0.938 | 0.674 | -0.020 |  |  |
| 6 | Southern Altiplano | high-profile | Topographic | 0.937 | 0.661 | -0.010 |  |  |
| 6 | Southern Altiplano | low-profile | Environment | 0.633 | 0.969 | -0.050 |  |  |
| 6 | Southern Altiplano | low-profile | Geographic | 1.111 | 0.281 | 0.050 |  |  |
| 6 | Southern Altiplano | low-profile | Topographic | 0.922 | 0.673 | -0.020 |  |  |
| 6 | Southern Altiplano | motile | Environment | 0.929 | 0.694 | -0.010 |  |  |
| 6 | Southern Altiplano | motile | Geographic | 1.095 | 0.199 | 0.042 |  |  |
| 6 | Southern Altiplano | motile | Topographic | 1.022 | 0.411 | 0.006 |  |  |
| 6 | Southern Altiplano | planktonic | Environment | 1.116 | 0.397 | 0.018 |  |  |
| 6 | Southern Altiplano | planktonic | Geographic | 1.629 | 0.095 | 0.224 |  |  |
| 6 | Southern Altiplano | planktonic | Topographic | 1.791 | 0.086 | 0.196 |  |  |

**Table S5.2** Results of variance partitioning showing the pure and shared fractions of variability (Adj. R^2^) explained by the environmental, geographical and topographical components in diatom species composition for the six lakes clusters identified through PCA and cluster analyses. Matrix: subset of diatom species data (all, and for each ecological guild separately: high-profile, low-profile, motile and planktonic).

| **Cluster** | **Name** | **Matrix** | **Predictors** | **Adj.R^2^** | **F** | ***p*** |
| --- | --- | --- | --- | --- | --- | --- |
| 1 | Ecuadorean-Colombian lowlands | all species | Environment | 0.029 | 1.270 | 0.013 |
| 1 | Ecuadorean-Colombian lowlands | all species | Geographic | 0.200 | 1.363 | 0.001 |
| 1 | Ecuadorean-Colombian lowlands | all species | Topographic | 0.009 | 1.231 | 0.115 |
| 1 | Ecuadorean-Colombian lowlands | all species | shared env+geo | 0.037 |  |  |
| 1 | Ecuadorean-Colombian lowlands | all species | shared geo+topo | 0.003 |  |  |
| 1 | Ecuadorean-Colombian lowlands | all species | shared topo+env | 0.000 |  |  |
| 1 | Ecuadorean-Colombian lowlands | all species | Shared total | 0.015 |  |  |
| 1 | Ecuadorean-Colombian lowlands | all species | residuals | 0.610 |  |  |
| 1 | Ecuadorean-Colombian lowlands | high-profile | Environment | 0.000 | 0.769 | 0.671 |
| 1 | Ecuadorean-Colombian lowlands | high-profile | Geographic | 0.164 | 2.348 | 0.001 |
| 1 | Ecuadorean-Colombian lowlands | high-profile | Topographic | 0.017 | 1.381 | 0.225 |
| 1 | Ecuadorean-Colombian lowlands | high-profile | shared env+geo | 0.112 |  |  |
| 1 | Ecuadorean-Colombian lowlands | high-profile | shared geo+topo | 0.000 |  |  |
| 1 | Ecuadorean-Colombian lowlands | high-profile | shared topo+env | 0.000 |  |  |
| 1 | Ecuadorean-Colombian lowlands | high-profile | Shared total | 0.140 |  |  |
| 1 | Ecuadorean-Colombian lowlands | high-profile | residuals | 0.593 |  |  |
| 1 | Ecuadorean-Colombian lowlands | low-profile | Environment | 0.000 | 0.725 | 0.839 |
| 1 | Ecuadorean-Colombian lowlands | low-profile | Geographic | 0.080 | 2.679 | 0.001 |
| 1 | Ecuadorean-Colombian lowlands | low-profile | Topographic | 0.019 | 1.397 | 0.122 |
| 1 | Ecuadorean-Colombian lowlands | low-profile | shared env+geo | 0.182 |  |  |
| 1 | Ecuadorean-Colombian lowlands | low-profile | shared geo+topo | 0.022 |  |  |
| 1 | Ecuadorean-Colombian lowlands | low-profile | shared topo+env | 0.025 |  |  |
| 1 | Ecuadorean-Colombian lowlands | low-profile | Shared total | 0.142 |  |  |
| 1 | Ecuadorean-Colombian lowlands | low-profile | residuals | 0.549 |  |  |
| 1 | Ecuadorean-Colombian lowlands | motile | Environment | 0.012 | 1.350 | 0.178 |
| 1 | Ecuadorean-Colombian lowlands | motile | Geographic | 0.043 | 1.503 | 0.059 |
| 1 | Ecuadorean-Colombian lowlands | motile | Topographic | 0.000 | 1.050 | 0.399 |
| 1 | Ecuadorean-Colombian lowlands | motile | shared env+geo | 0.033 |  |  |
| 1 | Ecuadorean-Colombian lowlands | motile | shared geo+topo | 0.096 |  |  |
| 1 | Ecuadorean-Colombian lowlands | motile | shared topo+env | 0.000 |  |  |
| 1 | Ecuadorean-Colombian lowlands | motile | shared total | 0.160 |  |  |
| 1 | Ecuadorean-Colombian lowlands | motile | residuals | 0.714 |  |  |
| 1 | Ecuadorean-Colombian lowlands | planktonic | Environment | 0.000 | 0.668 | 0.706 |
| 1 | Ecuadorean-Colombian lowlands | planktonic | Geographic | 0.340 | 4.919 | 0.001 |
| 1 | Ecuadorean-Colombian lowlands | planktonic | Topographic | 0.036 | 1.391 | 0.246 |
| 1 | Ecuadorean-Colombian lowlands | planktonic | shared env+geo | 0.182 |  |  |
| 1 | Ecuadorean-Colombian lowlands | planktonic | shared geo+topo | 0.000 |  |  |
| 1 | Ecuadorean-Colombian lowlands | planktonic | shared topo+env | 0.000 |  |  |
| 1 | Ecuadorean-Colombian lowlands | planktonic | shared total | 0.108 |  |  |
| 1 | Ecuadorean-Colombian lowlands | planktonic | residuals | 0.373 |  |  |
| 2 | Ecuadorean-Colombian Andes | all species | Environment | 0.009 | 1.192 | 0.022 |
| 2 | Ecuadorean-Colombian Andes | all species | Geographic | 0.053 | 1.659 | 0.001 |
| 2 | Ecuadorean-Colombian Andes | all species | Topographic | 0.000 | 0.953 | 0.602 |
| 2 | Ecuadorean-Colombian Andes | all species | shared env+geo | 0.000 |  |  |
| 2 | Ecuadorean-Colombian Andes | all species | shared geo+topo | 0.000 |  |  |
| 2 | Ecuadorean-Colombian Andes | all species | shared topo+env | 0.001 |  |  |
| 2 | Ecuadorean-Colombian Andes | all species | shared total | 0.014 |  |  |
| 2 | Ecuadorean-Colombian Andes | all species | residuals | 0.925 |  |  |
| 2 | Ecuadorean-Colombian Andes | high-profile | Environment | 0.008 | 1.130 | 0.285 |
| 2 | Ecuadorean-Colombian Andes | high-profile | Geographic | 0.084 | 2.046 | 0.002 |
| 2 | Ecuadorean-Colombian Andes | high-profile | Topographic | 0.000 | 0.825 | 0.625 |
| 2 | Ecuadorean-Colombian Andes | high-profile | shared env+geo | 0.057 |  |  |
| 2 | Ecuadorean-Colombian Andes | high-profile | shared geo+topo | 0.018 |  |  |
| 2 | Ecuadorean-Colombian Andes | high-profile | shared topo+env | 0.000 |  |  |
| 2 | Ecuadorean-Colombian Andes | high-profile | shared total | 0.080 |  |  |
| 2 | Ecuadorean-Colombian Andes | high-profile | residuals | 0.776 |  |  |
| 2 | Ecuadorean-Colombian Andes | low-profile | Environment | 0.000 | 0.579 | 0.822 |
| 2 | Ecuadorean-Colombian Andes | low-profile | Geographic | 0.047 | 3.352 | 0.009 |
| 2 | Ecuadorean-Colombian Andes | low-profile | Topographic |  |  |  |
| 2 | Ecuadorean-Colombian Andes | low-profile | shared env+geo | 0.052 |  |  |
| 2 | Ecuadorean-Colombian Andes | low-profile | shared geo+topo |  |  |  |
| 2 | Ecuadorean-Colombian Andes | low-profile | shared topo+env |  |  |  |
| 2 | Ecuadorean-Colombian Andes | low-profile | shared total | 0.052 |  |  |
| 2 | Ecuadorean-Colombian Andes | low-profile | residuals | 0.910 |  |  |
| 2 | Ecuadorean-Colombian Andes | motile | Environment | 0.026 | 1.620 | 0.071 |
| 2 | Ecuadorean-Colombian Andes | motile | Geographic | 0.098 | 3.359 | 0.001 |
| 2 | Ecuadorean-Colombian Andes | motile | Topographic |  |  |  |
| 2 | Ecuadorean-Colombian Andes | motile | shared env+geo | 0.047 |  |  |
| 2 | Ecuadorean-Colombian Andes | motile | shared geo+topo |  |  |  |
| 2 | Ecuadorean-Colombian Andes | motile | shared topo+env |  |  |  |
| 2 | Ecuadorean-Colombian Andes | motile | shared total | 0.047 |  |  |
| 2 | Ecuadorean-Colombian Andes | motile | residuals | 0.830 |  |  |
| 2 | Ecuadorean-Colombian Andes | planktonic | Environment | 0.025 | 1.416 | 0.078 |
| 2 | Ecuadorean-Colombian Andes | planktonic | Geographic | 0.078 | 2.920 | 0.001 |
| 2 | Ecuadorean-Colombian Andes | planktonic | Topographic | 0.000 | 0.750 | 0.675 |
| 2 | Ecuadorean-Colombian Andes | planktonic | shared env+geo | 0.053 |  |  |
| 2 | Ecuadorean-Colombian Andes | planktonic | shared geo+topo | 0.022 |  |  |
| 2 | Ecuadorean-Colombian Andes | planktonic | shared topo+env | 0.000 |  |  |
| 2 | Ecuadorean-Colombian Andes | planktonic | shared total | 0.062 |  |  |
| 2 | Ecuadorean-Colombian Andes | planktonic | residuals | 0.769 |  |  |
| 3 | Bolivian-Brazilian lowlands | all species | Environment | 0.000 |  |  |
| 3 | Bolivian-Brazilian lowlands | all species | Geographic | 0.049 | 1.529 | 0.013 |
| 3 | Bolivian-Brazilian lowlands | all species | Topographic | 0.053 | 1.576 | 0.014 |
| 3 | Bolivian-Brazilian lowlands | all species | shared | 0.000 |  |  |
| 3 | Bolivian-Brazilian lowlands | all species | residuals | 0.922 |  |  |
| 3 | Bolivian-Brazilian lowlands | high-profile | Environment | 0.126 | 1.777 | 0.004 |
| 3 | Bolivian-Brazilian lowlands | high-profile | Geographic | 0.054 | 0.679 | 0.799 |
| 3 | Bolivian-Brazilian lowlands | high-profile | Topographic | 0.012 | 1.125 | 0.311 |
| 3 | Bolivian-Brazilian lowlands | high-profile | shared env+geo | 0.041 |  |  |
| 3 | Bolivian-Brazilian lowlands | high-profile | shared geo+topo | 0.082 |  |  |
| 3 | Bolivian-Brazilian lowlands | high-profile | shared topo+env | 0.083 |  |  |
| 3 | Bolivian-Brazilian lowlands | high-profile | shared total | 0.000 |  |  |
| 3 | Bolivian-Brazilian lowlands | high-profile | residuals | 0.800 |  |  |
| 3 | Bolivian-Brazilian lowlands | low-profile | Environment | 0.000 |  |  |
| 3 | Bolivian-Brazilian lowlands | low-profile | Geographic | 0.000 | 0.940 | 0.509 |
| 3 | Bolivian-Brazilian lowlands | low-profile | Topographic | 0.047 | 1.530 | 0.117 |
| 3 | Bolivian-Brazilian lowlands | low-profile | shared | 0.064 |  |  |
| 3 | Bolivian-Brazilian lowlands | low-profile | residuals | 0.894 |  |  |
| 3 | Bolivian-Brazilian lowlands | motile | Environment | 0.044 | 1.357 | 0.032 |
| 3 | Bolivian-Brazilian lowlands | motile | Geographic | 0.000 |  |  |
| 3 | Bolivian-Brazilian lowlands | motile | Topographic | 0.048 | 1.483 | 0.069 |
| 3 | Bolivian-Brazilian lowlands | motile | shared | 0.045 |  |  |
| 3 | Bolivian-Brazilian lowlands | motile | residuals | 0.891 |  |  |
| 3 | Bolivian-Brazilian lowlands | planktonic | Environment | 0.000 |  |  |
| 3 | Bolivian-Brazilian lowlands | planktonic | Geographic | 0.000 |  |  |
| 3 | Bolivian-Brazilian lowlands | planktonic | Topographic | 0.165 | 2.071 | 0.048 |
| 3 | Bolivian-Brazilian lowlands | planktonic | shared | 0.000 |  |  |
| 3 | Bolivian-Brazilian lowlands | planktonic | residuals | 0.000 |  |  |
| 4 | Peruvian Andes | all species | Environment | 0.012 | 1.295 | 0.001 |
| 4 | Peruvian Andes | all species | Geographic | 0.033 | 1.376 | 0.001 |
| 4 | Peruvian Andes | all species | Topographic | 0.028 | 1.233 | 0.001 |
| 4 | Peruvian Andes | all species | shared env+geo | 0.011 |  |  |
| 4 | Peruvian Andes | all species | shared geo+topo | 0.008 |  |  |
| 4 | Peruvian Andes | all species | shared topo+env | 0.006 |  |  |
| 4 | Peruvian Andes | all species | shared total | 0.038 |  |  |
| 4 | Peruvian Andes | all species | residuals | 0.865 |  |  |
| 4 | Peruvian Andes | high-profile | Environment | 0.011 | 1.457 | 0.024 |
| 4 | Peruvian Andes | high-profile | Geographic | 0.024 | 1.681 | 0.002 |
| 4 | Peruvian Andes | high-profile | Topographic | 0.027 | 1.590 | 0.001 |
| 4 | Peruvian Andes | high-profile | shared env+geo | 0.002 |  |  |
| 4 | Peruvian Andes | high-profile | shared geo+topo | 0.016 |  |  |
| 4 | Peruvian Andes | high-profile | shared topo+env | 0.003 |  |  |
| 4 | Peruvian Andes | high-profile | shared total | 0.022 |  |  |
| 4 | Peruvian Andes | high-profile | residuals | 0.895 |  |  |
| 4 | Peruvian Andes | low-profile | Environment | 0.004 | 1.354 | 0.117 |
| 4 | Peruvian Andes | low-profile | Geographic | 0.026 | 2.075 | 0.001 |
| 4 | Peruvian Andes | low-profile | Topographic | 0.000 |  |  |
| 4 | Peruvian Andes | low-profile | shared | 0.006 |  |  |
| 4 | Peruvian Andes | low-profile | residuals | 0.965 |  |  |
| 4 | Peruvian Andes | motile | Environment | 0.041 | 1.161 | 0.037 |
| 4 | Peruvian Andes | motile | Geographic | 0.008 | 1.206 | 0.064 |
| 4 | Peruvian Andes | motile | Topographic | 0.039 | 1.480 | 0.001 |
| 4 | Peruvian Andes | motile | shared env+geo | 0.013 |  |  |
| 4 | Peruvian Andes | motile | shared geo+topo | 0.018 |  |  |
| 4 | Peruvian Andes | motile | shared topo+env | 0.002 |  |  |
| 4 | Peruvian Andes | motile | shared total | 0.012 |  |  |
| 4 | Peruvian Andes | motile | residuals | 0.904 |  |  |
| 4 | Peruvian Andes | planktonic | Environment | 0.015 | 2.297 | 0.091 |
| 4 | Peruvian Andes | planktonic | Geographic | 0.000 |  |  |
| 4 | Peruvian Andes | planktonic | Topographic | 0.107 | 2.681 | 0.001 |
| 4 | Peruvian Andes | planktonic | shared | 0.020 |  |  |
| 4 | Peruvian Andes | planktonic | residuals | 0.859 |  |  |
| 5 | Bolivian Andes | all species | Environment | 0.044 | 2.308 | 0.001 |
| 5 | Bolivian Andes | all species | Geographic | 0.212 | 1.928 | 0.001 |
| 5 | Bolivian Andes | all species | Topographic | 0.002 | 1.209 | 0.230 |
| 5 | Bolivian Andes | all species | shared env+geo | 0.055 |  |  |
| 5 | Bolivian Andes | all species | shared geo+topo | 0.004 |  |  |
| 5 | Bolivian Andes | all species | shared topo+env | 0.002 |  |  |
| 5 | Bolivian Andes | all species | shared total | 0.104 |  |  |
| 5 | Bolivian Andes | all species | residuals | 0.711 |  |  |
| 5 | Bolivian Andes | high-profile | Environment | 0.023 | 1.586 | 0.001 |
| 5 | Bolivian Andes | high-profile | Geographic | 0.026 | 2.975 | 0.001 |
| 5 | Bolivian Andes | high-profile | Topographic | 0.003 | 1.219 | 0.165 |
| 5 | Bolivian Andes | high-profile | shared env+geo | 0.001 |  |  |
| 5 | Bolivian Andes | high-profile | shared geo+topo | 0.005 |  |  |
| 5 | Bolivian Andes | high-profile | shared topo+env | 0.000 |  |  |
| 5 | Bolivian Andes | high-profile | shared total | 0.040 |  |  |
| 5 | Bolivian Andes | high-profile | residuals | 0.902 |  |  |
| 5 | Bolivian Andes | low-profile | Environment | 0.008 | 1.297 | 0.070 |
| 5 | Bolivian Andes | low-profile | Geographic | 0.017 | 1.436 | 0.011 |
| 5 | Bolivian Andes | low-profile | Topographic | 0.000 | 0.789 | 0.884 |
| 5 | Bolivian Andes | low-profile | shared env+geo | 0.015 |  |  |
| 5 | Bolivian Andes | low-profile | shared geo+topo | 0.040 |  |  |
| 5 | Bolivian Andes | low-profile | shared topo+env | 0.015 |  |  |
| 5 | Bolivian Andes | low-profile | shared total | 0.018 |  |  |
| 5 | Bolivian Andes | low-profile | residuals | 0.893 |  |  |
| 5 | Bolivian Andes | motile | Environment | 0.012 | 1.326 | 0.014 |
| 5 | Bolivian Andes | motile | Geographic | 0.029 | 1.740 | 0.001 |
| 5 | Bolivian Andes | motile | Topographic | 0.010 | 1.390 | 0.013 |
| 5 | Bolivian Andes | motile | shared env+geo | 0.017 |  |  |
| 5 | Bolivian Andes | motile | shared geo+topo | 0.041 |  |  |
| 5 | Bolivian Andes | motile | shared topo+env | 0.012 |  |  |
| 5 | Bolivian Andes | motile | shared total | 0.060 |  |  |
| 5 | Bolivian Andes | motile | residuals | 0.890 |  |  |
| 5 | Bolivian Andes | planktonic | Environment | 0.015 | 3.151 | 0.001 |
| 5 | Bolivian Andes | planktonic | Geographic | 0.000 |  |  |
| 5 | Bolivian Andes | planktonic | Topographic | 0.000 |  |  |
| 5 | Bolivian Andes | planktonic | shared | 0.000 |  |  |
| 5 | Bolivian Andes | planktonic | residuals | 0.000 |  |  |
| 6 | Southern Altiplano | all species | Environment | 0.034 | 1.209 | 0.175 |
| 6 | Southern Altiplano | all species | Geographic | 0.067 | 1.434 | 0.031 |
| 6 | Southern Altiplano | all species | Topographic | 0.000 |  |  |
| 6 | Southern Altiplano | all species | shared | 0.053 |  |  |
| 6 | Southern Altiplano | all species | residuals | 0.891 |  |  |
| 6 | Southern Altiplano | high-profile | Environment | 0.000 |  |  |
| 6 | Southern Altiplano | high-profile | Geographic | 0.000 |  |  |
| 6 | Southern Altiplano | high-profile | Topographic | 0.000 |  |  |
| 6 | Southern Altiplano | high-profile | shared | 0.000 |  |  |
| 6 | Southern Altiplano | high-profile | residuals | 0.000 |  |  |
| 6 | Southern Altiplano | low-profile | Environment | 0.000 |  |  |
| 6 | Southern Altiplano | low-profile | Geographic | 0.000 |  |  |
| 6 | Southern Altiplano | low-profile | Topographic | 0.000 |  |  |
| 6 | Southern Altiplano | low-profile | shared | 0.000 |  |  |
| 6 | Southern Altiplano | low-profile | residuals | 0.000 |  |  |
| 6 | Southern Altiplano | motile | Environment | 0.000 |  |  |
| 6 | Southern Altiplano | motile | Geographic | 0.000 |  |  |
| 6 | Southern Altiplano | motile | Topographic | 0.000 |  |  |
| 6 | Southern Altiplano | motile | shared | 0.000 |  |  |
| 6 | Southern Altiplano | motile | residuals | 0.000 |  |  |
| 6 | Southern Altiplano | planktonic | Environment | 0.000 |  |  |
| 6 | Southern Altiplano | planktonic | Geographic | 0.000 |  |  |
| 6 | Southern Altiplano | planktonic | Topographic | 0.000 |  |  |
| 6 | Southern Altiplano | planktonic | shared | 0.000 |  |  |
| 6 | Southern Altiplano | planktonic | residuals | 0.000 |  |  |
